# Guanylate‐binding proteins signature predicts favorable prognosis, immune‐hot microenvironment, and immunotherapy response in hepatocellular carcinoma

**DOI:** 10.1002/cam4.6347

**Published:** 2023-08-07

**Authors:** Yumei Ning, Shilin Fang, Jun Fang, Kun Lin, Haihang Nie, Peiling Xiong, Peishan Qiu, Qiu Zhao, Haizhou Wang, Fan Wang

**Affiliations:** ^1^ Department of Gastroenterology Zhongnan Hospital of Wuhan University Wuhan China; ^2^ Hubei Clinical Center and Key Lab of Intestinal and Colorectal Diseases Wuhan China; ^3^ Department of Infectious Disease Zhongnan Hospital of Wuhan University, Hubei AIDS Clinical Training Center Wuhan China; ^4^ Renmin Hospital of Huangmei County Huanggang China

**Keywords:** guanylate‐binding proteins, hepatocellular carcinoma, immunotherapy, tumor microenvironment

## Abstract

**Background:**

The role of guanylate‐binding proteins (GBPs) in various cancers has been elucidated recently. However, our knowledge of the clinical relevance and biological characteristics of GBPs in hepatocellular carcinoma (HCC) remains limited.

**Methods:**

A total of 955 HCC patients were enrolled from five independent public HCC cohorts. The role of GBP molecules in HCC was preliminarily investigated, and a GBP family signature, termed GBPs‐score, was constructed by principal component analysis to combine the GBP molecule values. We revealed the effects of GBP genes and GBPs‐score in HCC via well‐established bioinformatics methods and validated GBP1‐5 experimentally in a tissue microarray (TMA) cohort.

**Results:**

GBPs molecules were closely associated with the prognosis of patients with HCC, and a high GBPs‐score highly inferred a favorable survival outcome. We also revealed high GBPs‐score was related to anti‐tumor immunity, the immune‐hot tumor microenvironment (TME), and immunotherapy response. Among the GBPs members, GBP1‐5 rather than GBP6/7 may be dominant in these fields. The TMA analysis based on immunohistochemistry showed positive correlations between GBP1‐5 and the immune‐hot TME with abundant infiltration of CD8^+^ T cells in HCC.

**Conclusions:**

Our integrative study revealed the genetic and immunologic characterizations of GBPs in HCC and highlighted their potential values as promising biomarkers for prognosis and immunotherapy.

## 1 INTRODUCTION

Hepatocellular carcinoma (HCC) is one of the six most common cancers and the third leading cause of carcinoma death globally.^1^ Despite significant progress in surgery and targeted chemotherapy, the 5‐year survival rate for HCC is still unsatisfactory.^2^ Recently, with the development of the emerging immunotherapy, immune checkpoint inhibitors (ICIs) targeting programmed cell death protein 1 (PD‐1), programmed cell death ligand 1 (PD‐L1), and cytotoxic T lymphocyte antigen 4 (CTLA4), have been approved for a variety of cancers, including HCC.^3^, ^4^, ^5^, ^6^ However, more studies are still in progress due to the uncertain rates of remission and response.

It has been reported that the response rates of immunotherapy were associated with the complex tumor microenvironment (TME).^7^ Based on the immunologic patterns, tumors can be divided into “hot” and “cold” types. The “hot” tumor is often associated with an inflamed immune microenvironment with greater cytotoxic T‐lymphocyte (CTL) infiltration, active interferon (IFN)‐γ status, and sufficient antigen processing and presenting machinery (APM), which were pivotal factors for effective anti‐PD‐1/PD‐L1 therapy.^8^, ^9^ Conversely, the immune “cold” tumors display minimal CTL infiltrates, IFN‐γ desert, and antigen presentation defects, typically failing to respond to ICIs.^10^ Furthermore, the intratumoral cytolytic activity (CYT) was also an indicator of ICI response as it was strongly related to the CD8^+^ T cell infiltration and the expressions of genes involved in the activity of major histocompatibility complex class‐I (MHC‐I) APM.^11^ Due to the evident tumor heterogeneity between different patients, only a small percentage of patients with “hot” immune status show a response to ICIs.^9^ Therefore, identifying the immunologic pattern of the patient's TME may be beneficial to individualized therapy with ICIs.

Guanylate‐binding proteins (GBPs) are the family members of IFN‐γ inducible guanosine triphosphate hydrolases, consisting of GBP1‐7.^12^ Previous studies revealed GBPs play critical roles in innate immunity to defend against pathogens.^13^, ^14^ In addition, GBP molecules play complex roles in the progressions of tumors. For example, high expressions of GBP1, 2, and 5 with favorable prognoses were observed in patients with node‐negative breast cancer.^15^ And high expressions of GBP1‐5 were correlated with longer overall survival (OS) times in patients with skin cutaneous melanoma.^16^ Our previous study revealed that GBP2 exerted anti‐tumor effects in colorectal cancer (CRC) and may become a potential immunotherapy target.^17^ Nevertheless, highly expressed GBP1 was closely associated with worse prognosis in patients with oral cavity squamous cell carcinoma.^18^ However, the role of GBPs in HCC has rarely been reported. As IFN‐γ induced genes, the expressions of GBPs are highly increased by IFN‐γ in many cells, including NK cells, B cells, and T cells.^15^ Meanwhile, these cells can be activated and infiltrate into tumors under IFN‐γ stimulation.^15^ This accumulated evidence suggests GBP molecules play potential roles in TME.

In the present study, we aimed to comprehensively investigate the effects of seven GBP family molecules in HCC. The genetic characterizations and prognostic values of GBPs in HCC were preliminarily disclosed. To fully integrate the values of GBP molecules, GBPs‐score was constructed by the principal component analysis (PCA) algorithm. We demonstrated the favorable effects of GBPs‐score on prognosis, anti‐tumor immunity, inflamed immune infiltration, and predicting immunotherapy responses in HCC patients from five independent cohorts. We also revealed the effects of individual GBP molecules through public cohorts and a tissue microarray (TMA) cohort. Overall, our study provides a better understanding of the immunologic patterns and clinical relevance of GBPs in HCC, as well as the predictive value of the GBP family signature for immunotherapy responses across cancers.

## 2 MATERIALS AND METHODS

### 2.1 Dataset source and preprocessing

**FIGURE 1:**
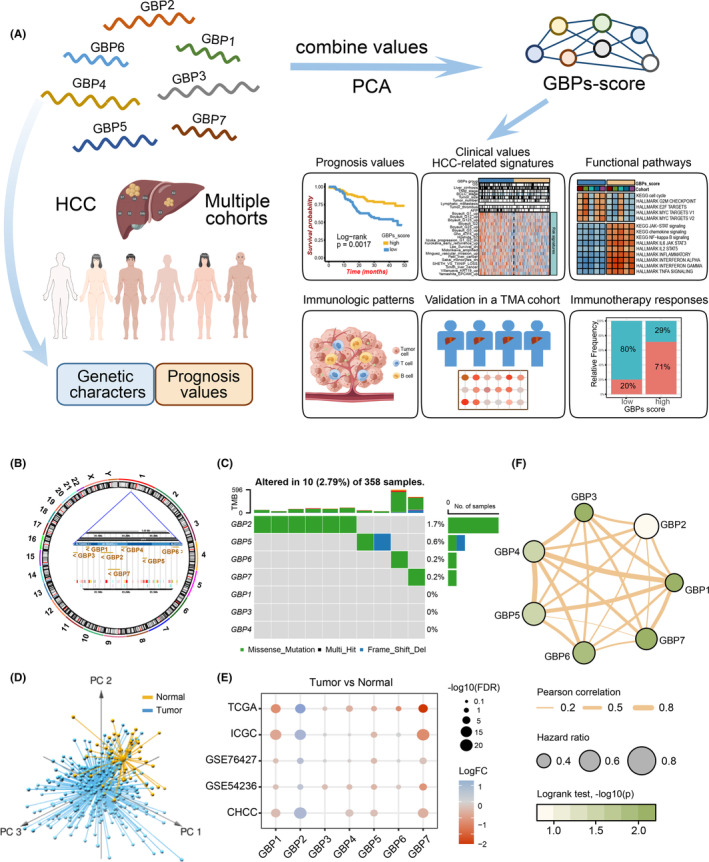
Landscape of genetic characterization of guanylate‐binding protein (GBP) family genes in hepatocellular carcinoma (HCC). (A) Overview of this work. Schematic diagrams of the human body and cells were created with the Figdraw website (www.figdraw.com). (B) The location of GBP family genes on human chromosomes. (C) The mutation frequency of GBPs in 358 patients of TCGA‐LIHC cohort. (D) Principal component analysis for the expression profiles of seven GBPs could distinguish tumors from normal samples in the TCGA‐LIHC cohort. (E) The differential expression of seven GBPs between liver cancer tissues and normal tissues in five independent cohorts. The expression profile of the CHCC cohort lacked the information of GBP3 and GBP6. (F) The prognostic values of seven GBPs and correlations between GBP members in the TCGA‐LIHC cohort.

The workflow of this study is displayed in Figure 1A. A total of 955 patients diagnosed with liver cancer were enrolled from five independent cohorts: TCGA (The Cancer Genome Atlas)‐LIHC, ICGC (International Cancer Genome Consortium)‐LIRI, GSE76427, GSE54236 and Chinese HCC (CHCC). For the TCGA‐LIHC and ICGC‐LIRI cohorts, RNA sequencing data (FPKM) were downloaded and log2 transformed from UCSC Xena (https://xenabrowser.net/datapages/) and the ICGC database (https://icgc.org/), respectively. GSE76427 and GSE54236 were retrieved from Gene Expression Omnibus (GEO) database (http://wwwncbinlmnih.gov/geo/). Gene expression profile and clinical information of the CHCC cohort were obtained directly from the supplementary materials of the published article.^19^

Moreover, eight immunotherapy groups were recruited to test the predictive value of GBPs‐score responses to immunotherapy: The IMvigor210 cohort (bladder cancer, anti‐PD‐L1) was obtained from http://research‐pub.Gene.com/imvigor210corebiologies/.^20^ GSE35640 (melanoma and non‐small‐cell lung cancer, MAGE‐A3) and GSE126044 (non‐small‐cell lung cancer, anti‐PD‐1) were downloaded from the GEO. Five additional immunotherapy cohorts containing detailed RNA expression matrixes and clinical information were retrieved from the TIDE website (http://tide.dfci.harvard.edu/download/).^21^ Table S1 summarizes the cohorts included in this study.

### 2.2 Construction of GBPs‐score

To combine the values of GBP family genes and evaluate them in individual patients with HCC, we constructed a scoring system that we termed GBPs‐score. First, PCA was conducted. Secondly, the first two principal components (PC1 and PC2) were selected to construct the GBPs‐score. This approach has the advantage of focusing the score on the most well‐related (or anti‐related) genes to integrate the gene contributions fully.^22^ Thirdly, we define the GBPs‐score using a method similar to previous studies^23^:

$$\text{GBPs}-\text{score}=\sum \;\left[\left(\text{PC1}+\text{PC2}\right)\times \text{Expi}\right]$$

where Expi is the expression of GBP family genes.

### 2.3 Tissue microarrays cohort and immunohistochemistry (IHC)

HCC TMA cohort (HLivH180Su11) consisting of 94 HCC tissues was supplied by Shanghai Outdo Biotech together with detailed clinical information (Table S2). Written informed consent was obtained from all enrolled subjects in accordance with the guidelines of the Declaration of Helsinki. This TMA cohort was used to evaluate the role of GBP1‐5 in HCC prognosis and establish the relationships of GBP1‐5 with CD8 and PD‐L1 expression. IHC staining was performed using the two‐step method of Dako Envision™ Detection System (DakoCyto‐mation, Denmark). Primary antibodies against GBP1‐5 (1:500, Santa Cruz Biotechnology, Inc, sc‐166960), CD8 (PA067, Abcarta), PD‐L1 (PA167, Abcarta) were used. Protein expression was determined by positive cell rate. The IHC score of GBP1‐5 was calculated by multiplying the staining intensity grade (indicating negative, weak‐positive, moderate‐positive, and strong‐positive at grades 0, 1, 2, and 3, respectively) with the positive cell rate. The evaluation was carried out independently by two observers.

### 2.4 Statistical analysis

All analyses were conducted using GraphPad (version 8.0), R (version 4.1.1), and SPSS (version 25) software. The Student's *t*‐test and Wilcoxon rank‐sum test were selected as the standard tests. The relationships between GBPs‐score and other continuous variables were assessed by the Pearson method. The correlation between GBPs‐score subgroups and clinical features was analyzed by the *χ* ^2^ test. A two‐tailed *p* value < 0.05 was considered as statistical significance.

Other bioinformatics methods are detailed in the supplementary materials.

## 3 RESULTS

### 3.1 Landscape of the genetic characterization of GBP family genes in HCC

As shown in Figure 1B, all GBP family genes are located on human chromosome 1. Of the 358 samples of the TCGA‐LIHC cohort, 10 experienced mutations of GBPs with a frequency of 2.79%. Among the GBP family genes, GBP2 exhibited the highest mutation frequency, while GBP1, GBP3, and GBP4 did not show any mutations in the LIHC cohort (Figure 1C). In addition, HCC samples could be distinguished from the normal based on the expressions of these seven GBP genes in all five cohorts (Figure 1D; Figure S1). According to the cumulative variance plots, we observed the first and second principal components accounted for more than 60% of the contribution (Figures S1 and S2A). And the importance of each GBP molecule in the PCA was different (Figure S2B–F). Thus the first two principal components will be selected to construct the follow‐up GBPs signature. Furthermore, the expression of GBPs between HCC and normal samples was analyzed. Of note, only GBP2 expression was highly increased in HCC tissues compared with the normal, whereas GBP1, GBP5, and GBP7 expressions were significantly downregulated, and no significant differences were found in the remaining family members across the five cohorts (Figure 1E; Table S3). The GBP network presented a comprehensive landscape of the correlations and prognostic value of GBPs in the TCGA‐LIHC cohort (Figure 1F). Out of them, GBP1, GBP4, and GBP5 had the strongest positive correlations with each other. In addition, the correlations between GBP genes in the other four cohorts were displayed in Figure S3A–D.

### 3.2 Relationship between the GBP family genes and prognosis of HCC patients

**FIGURE 2:**
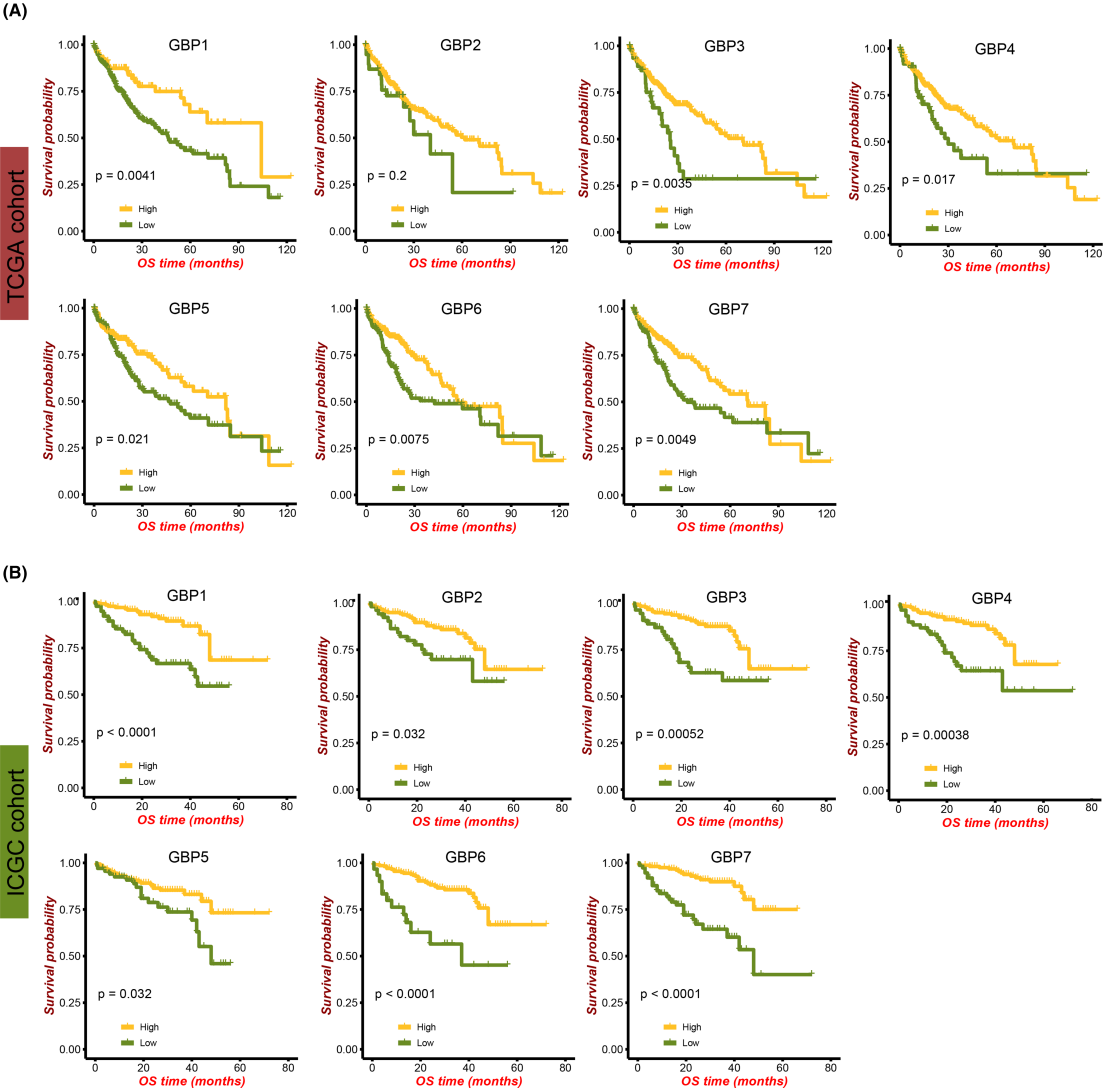
Correlations between guanylate‐binding protein1‐7 expression and prognosis of hepatocellular carcinoma patients in the TCGA (A) and ICGC (B) cohorts.

Next, to preliminarily investigate the prognostic values of GBP family molecules in HCC, Kaplan–Meier (K‐M) survival analysis was performed, using the “survminer” R package to determine the optimal cutoff. In the TCGA cohort, patients with high expression of GBP molecules, except for GBP2 (*p* = 0.12), were significantly associated with better survival outcomes (Figure 2A). In the ICGC cohort, all the GBP1‐7 molecules were favorable prognostic factors for HCC as their high expressions inferred longer overall survival times (Figure 2B). Moreover, the relationships between GBP molecules and HCC prognosis in the other three cohorts were displayed in Figure S4, showing similar results to the above. These data suggested that elevated GBP family expression was associated with a better prognosis of liver cancer on an overall trend, and GBPs may be potential prognostic biomarkers for HCC.

### 3.3 Construction of GBPs‐score and verification of its prognostic value in HCC

**FIGURE 3:**
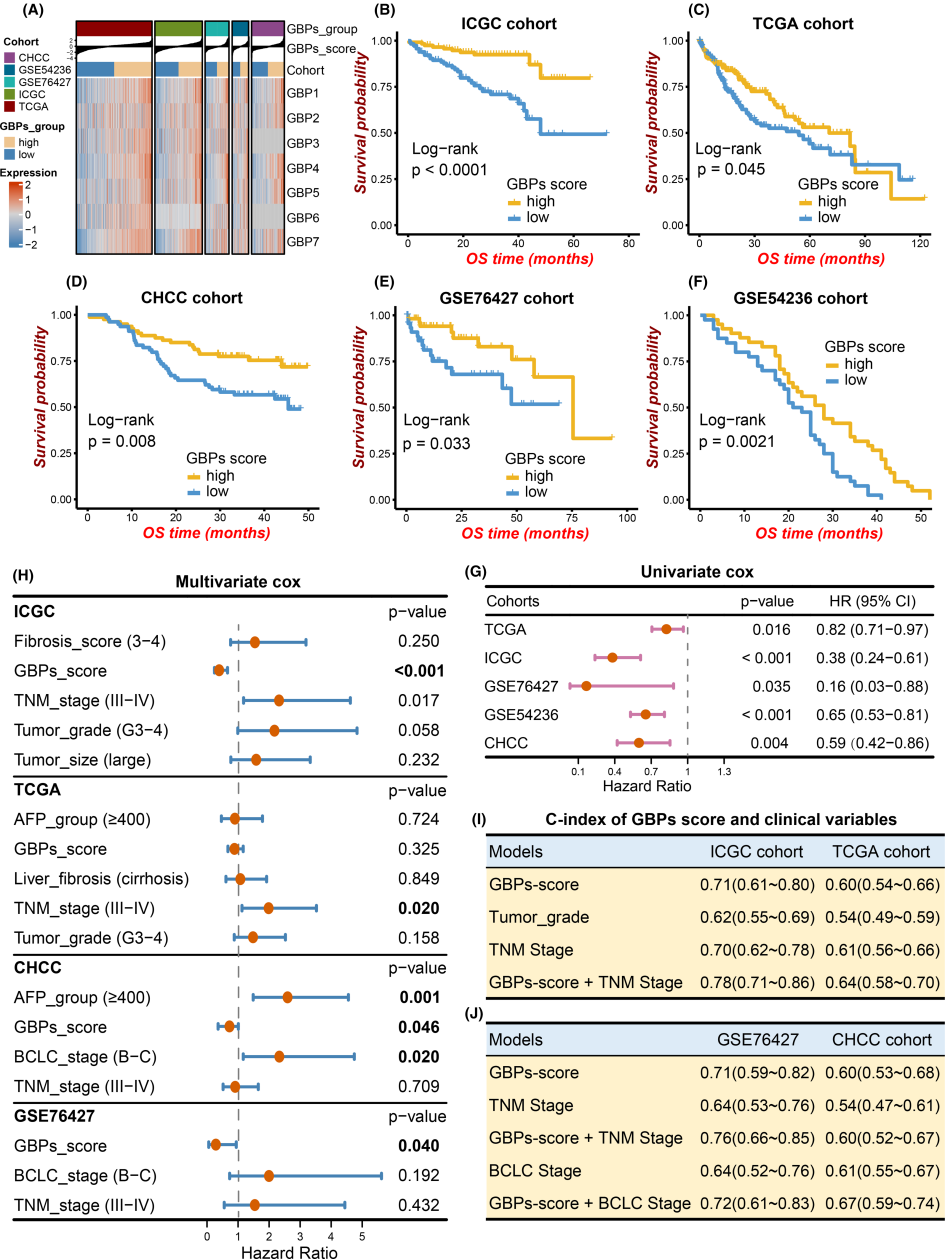
Prognostic values of guanylate‐binding proteins (GBPs)‐score in five independent cohorts. (A) Correlation between GBPs‐score and expression of GBPs across five cohorts. (B)–(F) Kaplan–Meier analysis showed the relationship between GBPs‐scores and the overall survival (OS) of hepatocellular carcinoma (HCC) samples in ICGC (B), TCGA (C), CHCC (D), GSE76427 (E), and GSE54236 (F) cohorts. (G) The forest plot showed the univariate Cox regression analysis for GBPs‐score in five HCC cohorts. (H) The forest plot showed the multivariate Cox regression analysis for GBPs‐score in the ICGC, TCGA, and CHCC cohorts. (I) and (J) The concordance indexes (C‐indexes) of clinical variates and GBPs‐score in ICGC and TCGA (I), GSE76427 and CHCC (J) cohorts.

Based on the above results, we speculated that GBP family molecules may have effects on the progression of HCC and may become a potential signature for predicting HCC prognosis. In order to further explore and better combine the value of GBP family genes, we adopted the PCA algorithm mentioned above and constructed a GBPs‐score based on the expressions of seven GBPs. This method has the advantage of concentrating the score on the most well‐related (or anti‐related) genes so that it fully integrates the gene contributions.^22^ The HCC samples from each cohort were divided into high and low GBPs‐score groups by the median GBPs‐scores (Figure 3A; Table S4). The baseline clinical characteristics between GBPs‐score subgroups were balanced, which were exhibited in Tables S5–S8. Subsequently, K‐M analysis showed that high GBPs‐scores were related to better survival outcomes in all five independent cohorts (Figure 3B–F). And univariate Cox regression further revealed that a high GBPs‐score was a favorable prognostic factor in all five cohorts (Figure 3G). Considering that multiple factors affect survival (such as TNM stage, tumor grade, tumor size, AFP level, BCLC stage, and liver cirrhosis), the multivariate Cox regression analysis was performed. Due to a lack of relevant clinical information, this analysis was not completed in the GSE54236 cohort. Notably, a higher GBPs‐score was significantly associated with the favorable prognosis in the ICGC, CHCC, and GSE76427 cohorts (Figure 3H; Table S9). The same trend was shown in the TCGA cohort, even though it was not statistically significant. Additionally, the C‐index was also used to quantify the predictive accuracy of the GBPs‐score for HCC prognosis. An integration of GBPs‐score and TNM stage/BCLC stage showed improved prognostic predictive ability compared with TNM/BCLC stage alone in the ICGC, TCGA, GSE76427, and CHCC cohorts (Figure 3I,J). The time‐dependent C‐indexes were consistent with these results (Figure S5). Taken together, these data suggested that the GBPs‐score may be a promising prognostic predictor for HCC and high scores inferred favorable outcomes of HCC patients.

### 3.4 High GBPs‐score was associated with favorable clinical features and favorable signatures of HCC‐related molecular classification

**FIGURE 4:**
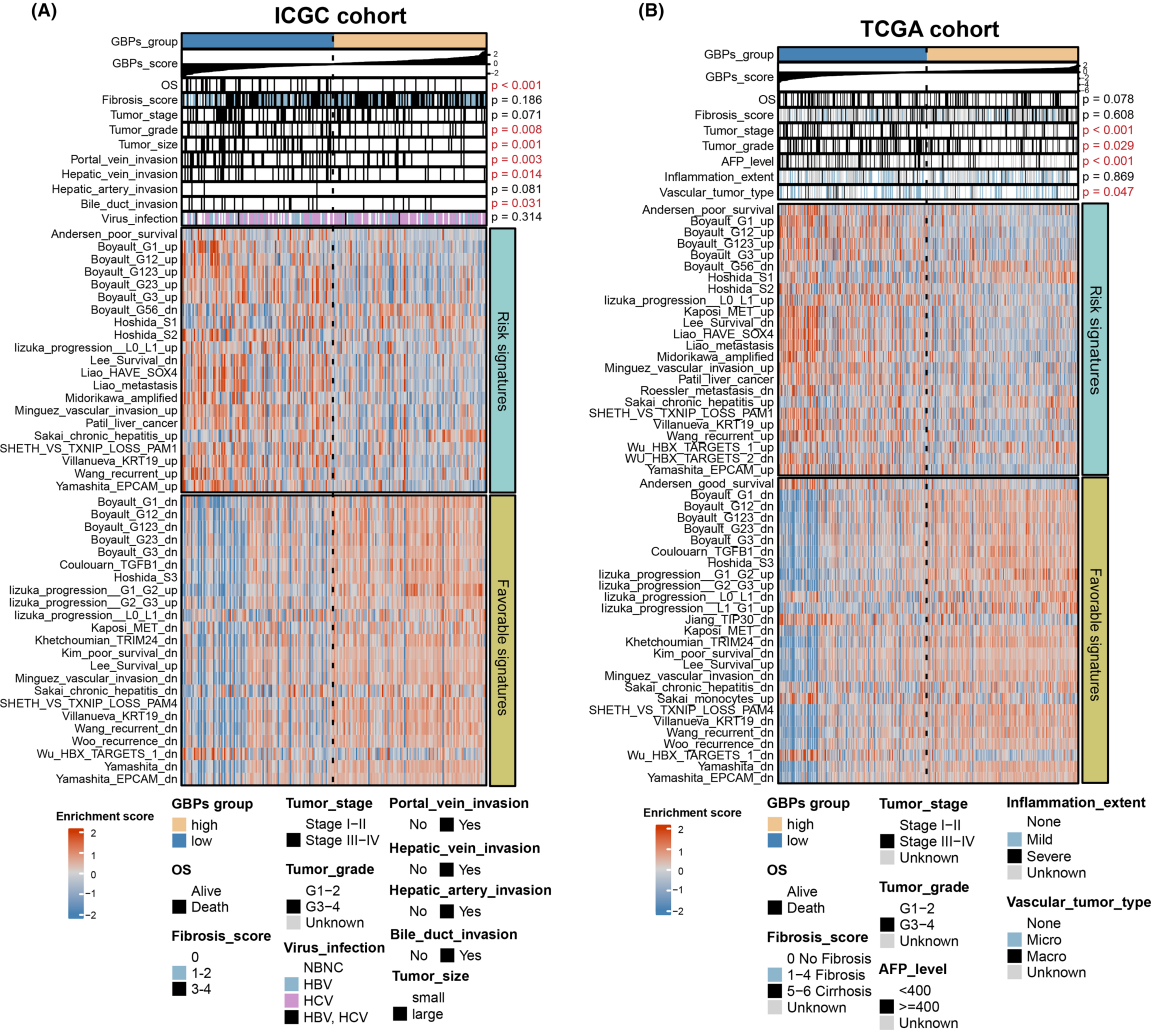
Relationships between guanylate‐binding proteins‐score and clinical features and other hepatocellular carcinoma‐related molecular signatures in the ICGC (A) and TCGA (B) cohorts.

Next, we explored the clinical characteristics and other HCC‐related molecular signatures based on GBPs‐score. Since the ICGC, TCGA, and CHCC cohorts had relatively complete clinical information, we used these three cohorts for further analysis. The results displayed the patients in the low GBPs‐score group were associated with worse TNM stage and grade, larger tumor size, and higher percentages of tumor invasion in the ICGC cohort (Figure 4A; Table S10). In the TCGA cohort, patients in the low GBPs‐score group were closely related to worse TNM stage and grade, higher AFP level, and higher percentages of tumor invasion and virus infection (Figure 4B; Table S11). In the CHCC cohort, a low GBP score was associated with higher AFP levels but not correlated with other clinical features (Figure S6; Table S12). In these three cohorts, no significant differences were found in liver cirrhosis or fibrosis score between GBPs subgroups. Taken together, GBPs were positively associated with favorable clinical features in HCC on an overall trend.

In addition, we compared GBPs‐score with other HCC‐related favorable or risk molecular signatures according to previous research.^19^ The heatmaps showed that the enrichment scores of signatures with favorable prognosis were significantly increased in the high GBPs‐score groups. In contrast, those with poor prognoses were significantly increased in the low GBPs‐score groups across the three cohorts (Figure 4A,B; Figure S6). Collectively, these data suggested that high GBPs‐score may be closely related to the favorable clinical features and signatures of HCC‐related molecular classification, but further studies are needed to investigate the correlation between GBPs‐score and clinical characteristics of HCC.

### 3.5 GBPs‐score was positively correlated with immune response and anti‐tumor immunity

**FIGURE 5:**
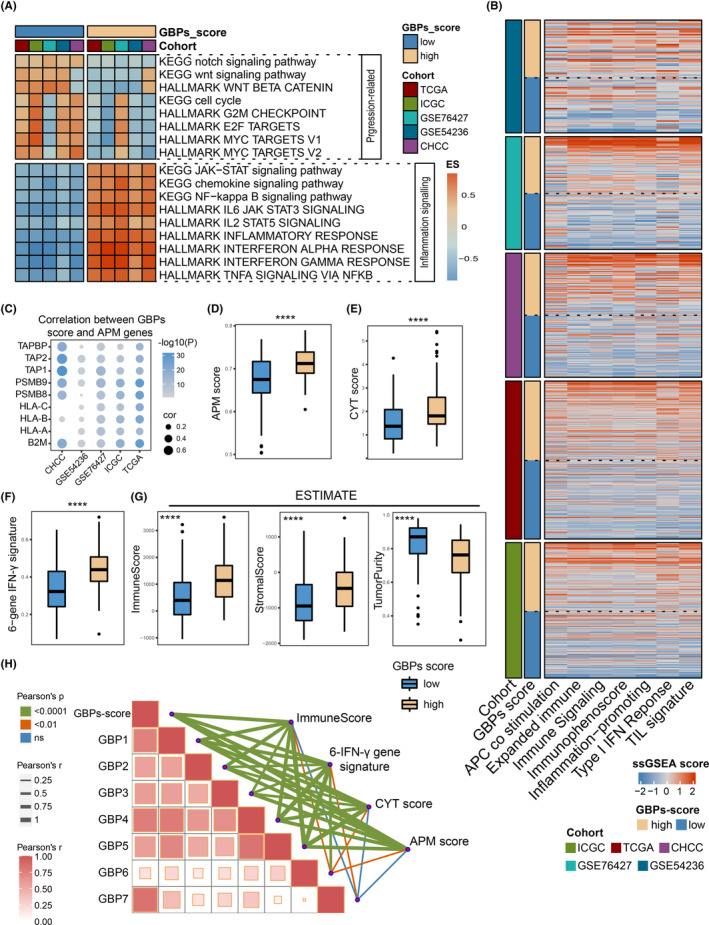
Correlation between guanylate‐binding proteins (GBPs)‐score and hepatocellular carcinoma (HCC) immune microenvironment. (A) GSEA revealed the differences in tumor progression‐related pathways and inflammation signaling pathways between GBPs‐score subgroups in five cohorts. (B) Differences of immunologic patterns between GBPs‐score subgroups. (C) Bubble plot showed the correlations between GBPs‐scores and HLA‐I antigen processing and presenting machinery (APM) genes in HCC samples of the five cohort. (D)–(F) The APM score (D), cytolytic activity (CYT score) (E), and 6‐gene interferon (IFN)‐γ signature (F) were calculated by ssGSEA between high and low GBPs‐score groups in ICGC cohort. (G) Immune score (left), stromal score (median), and tumor purity (right) were calculated by ESTIMATE between GBPs‐score subgroups in the ICGC cohort. (H) A correlation heat map illustrated the relationships among GBPs‐score and APM score, CYT score, 6‐gene IFN‐γ signature and immune score in HCC samples of the ICGC cohort.

To explore the biological behaviors between GBPs‐score subgroups, GSEA was conducted in the five cohorts (Figure 5A; Table S13). Low GBPs‐score samples showed marked enrichment in cancer progression‐related pathways such as Notch and WNT signaling pathways. They were also characterized by elevated “cell cycle”, “G2M checkpoints” and “E2F and MYC targets” signatures, suggesting proliferative characteristics for this subgroup. On the contrary, the high GBPs‐score group was distinguished by more inflamed characteristics, including JAK–STAT, chemokine, NF‐ kappa B signaling, inflammatory response, and IL‐, IFN‐, TNF‐related signaling pathways, with the IFN‐α/γ response being the most significant.

As an IFN‐γ stimulated family, GBP molecules are closely related to immune responses.^24^ Therefore, we further explored the effect of GBPs‐score on the immune microenvironment of HCC. The heatmap revealed that the samples with high GBPs‐scores had higher enrichment scores of signatures related to the immune response in the five HCC cohorts, indicating high GBPs‐score was associated with a “hot” immune status (Figure 5B). The APM is the first step in eliciting an effective anti‐tumor response in the body.^25^ Here, we found GBPs‐score was remarkably positively correlated with the expression of APM molecules in the five HCC cohorts (the expression profile of the CHCC cohort lacked HLA‐A and HLA‐C) (Figure 5C). Also, patients with high GBPs‐scores had higher APM scores, which were calculated by ssGSEA algorithm (Figure 5D; Figure S7A). The number of MHC‐I‐associated neoantigens was influenced by CYT, and high CYT can improve immune responses.^26^ In our study, the CYT score was significantly higher in the high GBPs‐score groups of the five cohorts (Figure 5E; Figure S7B), indicating the enhanced CYT in the high GBPs‐score samples. In addition, GBPs‐score was positively correlated with the 6‐gene IFN‐γ signature, which was used to reflect the IFN‐γ response (Figure 5F; Figure S7C).^27^ IFN‐γ is the most important cytokine implicated in anti‐tumor immunity.^28^ These data suggested high GBPs‐score was associated with the enhanced immune response in HCC. Further after, the ESTIMATE algorithm was used to evaluate the immune score, stromal score, and tumor purity. We observed patients with high GBPs‐scores had higher immune and stromal scores yet lower tumor purity (Figure 5G; Figure S8), demonstrating that high GBPs‐score may play a beneficial role in immune activation and anti‐tumor immunity. Notably, among GBP members, the expressions of GBP1‐5 were more strongly correlated with the above signature/scores (APM score, CYT score, 6‐gene IFN‐γ signature, and immune score), while GBP6‐7 had relatively weak correlations with them (Figure 5H; Table S14). These data were based on the ICGC cohort, with consistent results for the remaining four cohorts (Figure S9; Table S14). Together, these data suggested less tumor growth and a more inflamed microenvironment in HCC patients with high GBPs‐scores.

### 3.6 High GBPs‐score was associated with an immune‐hot TME with abundant immune cell infiltration

**FIGURE 6:**
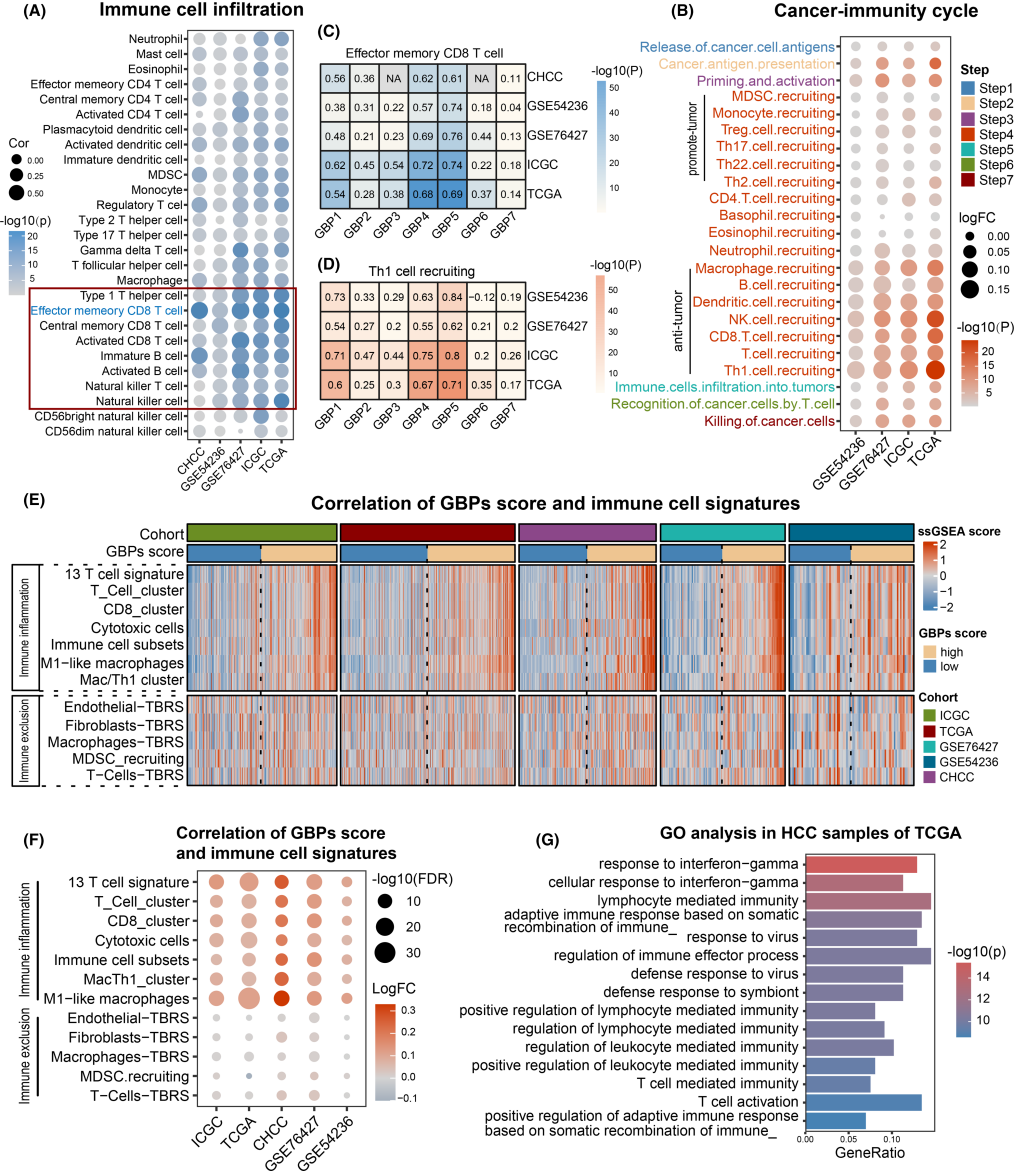
Tumor microenvironment cell infiltration characteristics in guanylate‐binding proteins (GBPs)‐score subgroups. (A) and (B) The correlations between GBPs‐score and immune cells (A), various steps of the cancer‐immunity cycle (B) in hepatocellular carcinoma (HCC) samples. (C)and (D) The correlations between GBP molecules and effector memory CD8^+^ T cells (C), Th1 cell recruiting (D) in HCC samples of five cohorts. (E) Heatmap showed the distinct immune‐related components of the GBPs‐score subgroups in five cohorts. (F) Bubble plot representation revealed the correlations of GBPs‐score and immune‐related components more visually. (G) GO analysis based on the top 200 differential expressed genes (high vs. low GBPs‐score) in the TCGA‐LIHC cohort.

Next, we explored the relationship of GBPs‐score with the immune cell infiltration of TME in HCC. We found that GBPs‐score was significantly positively correlated with the infiltration of a variety of immune cells, indicating that GBP molecules could participate in shaping an immune‐hot TME (Figure 6A; Table S15). Notably, the effector memory CD8^+^ T cell infiltration had the strongest positive association with GBPs‐score across all five cohorts (Figure 6A). As expected, GBPs‐score was highly associated with pivotal steps of the cancer‐immunity cycle, such as step 2 (cancer antigen presentation), step 3 (priming and activation), step 6 (recognition of cancer cells by T cells), and step 7 (killing of cancer cells) in four cohorts (Figure 6B; Table S16). For individual GBP members, GBP1 to 5 showed relatively strong positive correlations with the effector memory CD8^+^T cells, whereas GBP7 had no significant correlation with it (Figure 6C). Similarly, GBP1‐5 showed strong positive associations with the Th1 cell recruiting in the cancer‐immunity cycle, while GBP6‐7 had weak correlations with this step (Figure 6D). From these data, we observed that the GBPs‐score was positively correlated not only with the anti‐tumor cells that play roles in immune activation and tumor killing (such as CD8 T cells, Th1 cells, NK(T) cells, and B cells), but also with some immunosuppression‐related cells (such as Tregs and MDSC). Even so, we suggested that the improvement of GBPs‐score was mainly related to the activation of anti‐tumor immunity as the higher positive correlation between GBPs‐score and the antitumor cell infiltration, which was more obvious in the cancer‐immunity cycle. To further verify this result, we used single‐cell transcriptome data for analysis (Figure S10). The results showed that the expression of GBP1‐5 in CD8 T cells was higher than in Tregs, except for GBP2. The single‐cell data also revealed that GBP6 was not expressed in the liver and the expression of GBP7 in liver cancer immune cells was extremely low.

Moreover, other publicly available gene signatures that characterize immune infiltration were collected to further validate the immunologic patterns between GBPs‐score subgroups. Across the five cohorts, high enrichment scores of signatures reflecting immune inflammation were observed in the high GBPs‐score groups, such as 13 T cell signature, T cell and CD8 cluster, cytotoxic cells, immune cell subsets, M1‐like macrophages and Mac/Th1 cluster (Figure 6E). However, the signatures of immune exclusion, including endothelial‐, fibroblasts‐, macrophages‐, T cell‐TBRS (TGF‐beta response signatures), and MDSC (myeloid‐derived suppressor cells) recruiting, showed no significant differences between GBPs‐score subgroups (Figure 6E). The bubble plot representation revealed the results more visually (Figure 6F; Table S17). Additionally, GO enrichment analysis showed that patients with high GBPs‐scores were significantly enriched in lymphocyte‐ and T cell‐related (such as T cell activation, T cell, and lymphocyte‐mediated immunity), as well as other immune‐related functions (Figure 6G; Figure S11; Table S18).

**FIGURE 7:**
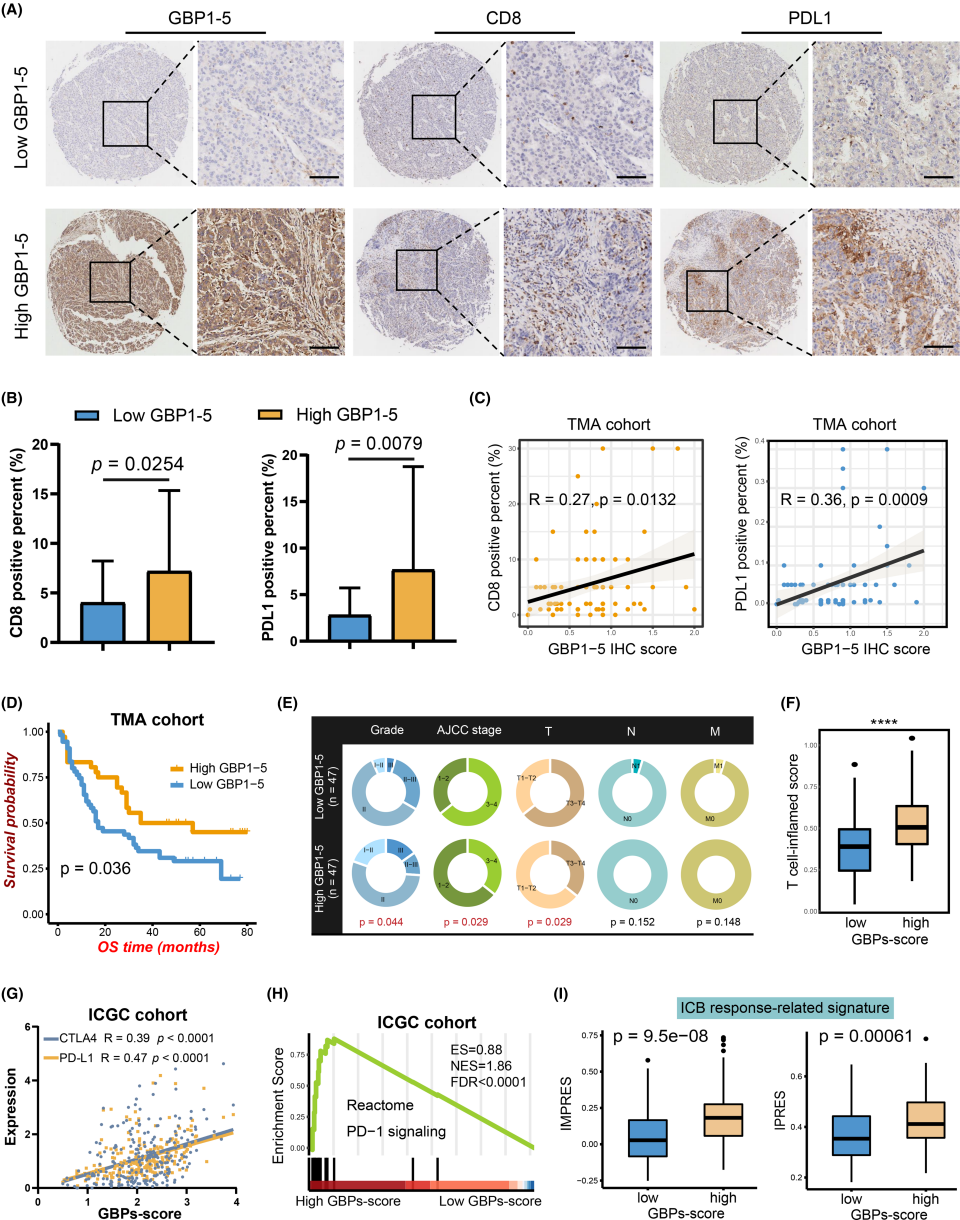
High guanylate‐binding proteins (GBPs)‐score potentially benefited to immunotherapeutic response. (A) Representative images of GBP1‐5, CD8, and programmed cell death ligand 1 (PD‐L1) immunohistochemistry (IHC) staining in hepatocellular carcinoma (HCC) samples were shown (scale bars: 100 μm). (B) Box plots showing the positive percent of CD8 (left) and PD‐L1 (right) between the high and low GBP1‐5 expression group of the HCC samples of tissue microarray (TMA) cohort. (C) Correlation between the GBP1‐5 IHC score and CD8 (left) and PD‐L1 (right) positive percent detected using immunofluorescence. (D) K‐M‐analysis of overall survival of HCC patients based on GBP1‐5 expression (*n* = 92). (E) Relationships between GBPs‐score and clinical features in the TMA cohort. (F) ssGSEA algorithm validated that the T cell‐inflamed score was significantly elevated in the high GBPs‐score group in HCC samples of the ICGC cohort. (G) The correlation between GBPs‐score and PD‐L1 and cytotoxic T lymphocyte antigen 4 in ICGC cohort. (H) GSEA plots of Reactome programmed cell death protein 1 signaling showed positive correlation with higher GBPs‐score in ICGC cohort. (I) Box plots showing expression of immune checkpoint blockade (ICB) response‐related signatures between GBPs‐score subgroups in the ICGC cohort.

GBP6 is reportedly constitutively expressed only in the oropharyngeal tract, and the RNA‐ sequencing transcriptome data showed its expression in HCC tissues was indeed extremely low (Figure S12). Besides, the above analyses demonstrated that GBP6,7 had a very weak association with immune activation, whereas GBP1‐5 had a strong association. Hence, GBP1‐5 caught our attention as it may play a more important role in immune activation. Experimentally, we verified the GBP1‐5 expression in a TMA cohort. Ninety‐two HCC samples were included for subsequent analysis according to the IHC staining of GBP1‐5 proteins (Table S19). We found that higher CD8 and PD‐L1 expression were shown in the high GBP1‐5 group (Figure 7A,B), and CD8 and PD‐L1 expression were positively correlated with GBP1‐5 (Figure 7C). Importantly, patients with higher GBP1‐5 expressions had better OS outcomes (Figure 7D). Additionally, high GBP1‐5 expression was related to a better AJCC stage, lower pathology grade, and smaller tumor size (Figure 7E). Taken together, these results indicated that a high GBPs‐score was correlated with CD8^+^ T cell infiltration, shaping an immune‐hot microenvironment in HCC.

### 3.7 High GBPs‐score potentially benefited immunotherapeutic response

**FIGURE 8:**
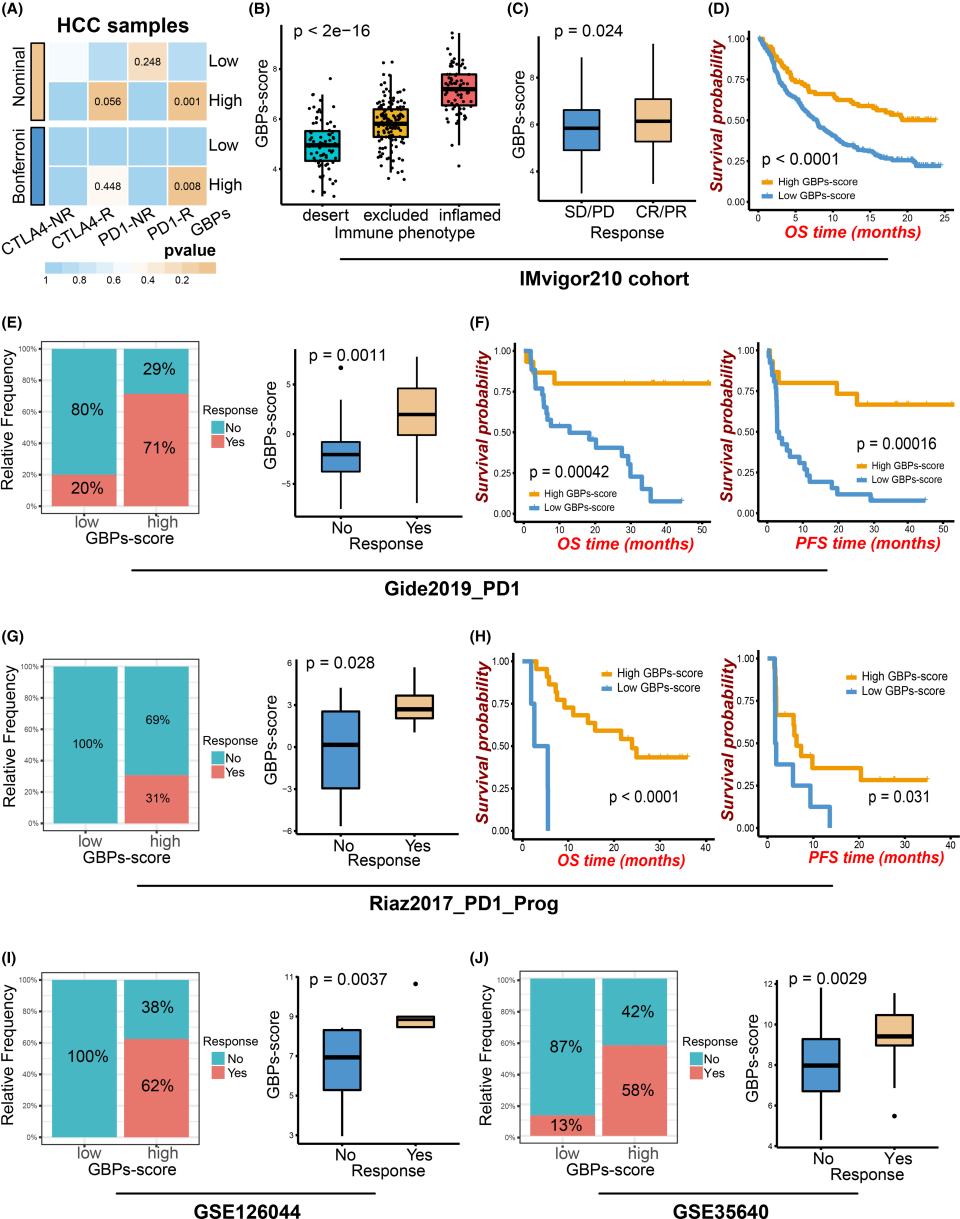
The role of guanylate‐binding proteins (GBPs)‐score in immunotherapy across cancers. (A) SubMap analysis revealed that the high GBPs‐score group exhibited a high likelihood of response to anti‐PD1 in the hepatocellular carcinoma samples of GSE76427. (B) Differences in GBPs‐score among distinct tumor immune phenotypes in the IMvigor210 cohort (bladder cancer). (C) GBPs‐score was higher in the anti‐programmed cell death ligand 1 partial/complete response (PR/CR) patients than the stable/progressive disease (SD/PD) in IMvigor210 cohort. (D) Survival analysis for GBPs‐score in the IMvigor210 cohort using Kaplan–Meier (K‐M) curves. Correlation of GBPs‐score and anti‐programmed cell death protein 1 response in Gide2019 (melanoma) (E), Riaz2017 (melanoma) (G), and GSE126044 (non‐small‐cell lung cancer) cohorts (I). Boxplots showed that GBPs‐scores were higher in responders and bar charts showed proportions of responders were more in high GBPs‐score groups. K‐M curves showed the relationships between GBPs‐score and the overall survival (OS) (left) and progression‐free survival (right) of patients in Gide2019 (F) and Riaz2017 cohorts (H). (J) Correlation of GBPs‐score and MAGE‐A3 response in GSE35640 (melanoma and non‐small‐cell lung cancer).

As the above conclusions suggested that GBP molecules were associated with immune‐hot TME in HCC, we next investigated the role of GBPs‐score in immunotherapy response. A T cell‐inflamed gene expression profile (GEP) which is correlated with immune checkpoint blockade (ICB) response was introduced into the analysis.^29^ As shown in the boxplots (Figure 7F; Figure S13A), the ssGSEA algorithm revealed that the T cell‐inflamed scores were significantly elevated in the high GBPs‐score groups of the four cohorts. Then we evaluated the critical immune checkpoints and found that the expression of PD‐L1 and CTLA4 elevated with increased GBPs‐score (Figure 6G; Figure S13B). GSEA revealed high GBPs‐scores were associated with the activation of Reactome PD‐1 signaling (Figure 7H; Figure S13C). In addition, higher enrichment scores of innate anti‐PD‐1 resistance and immune‐predictive score signatures were found in the high GBPs‐score groups (Figure 7I; Figure S13D). Furthermore, the SubMap analysis indicated the high GBPs‐score group had a high likelihood of response to anti‐PD1 treatment in patients with HCC (Figure 8A; Figure S14). This evidence suggested that patients with high GBPs‐scores can be more sensitive to immunotherapy.

We also explored the effect of GBPs‐score in the immunotherapy response of other cancers (including bladder cancer, gastric cancer, melanoma, and non‐small cell lung cancer) from eight immunotherapy‐related cohorts (six ICB cohorts, one adoptive T cell therapy cohort, and one MAGE‐A3 therapy cohort). In the IMvigor210 cohort, a higher GBPs‐score was significantly associated with the inflamed immune phenotype (Figure 8B), in which ICIs were easier to exert anti‐tumor effects. Across these cohorts, we observed substantially higher ICB response rates in the high GBPs‐score groups and higher GBPs‐scores in the responders (Figure 8C,E,G,I; Figure S15A,E,G). High GBPs‐score also predicted better prognoses of these patients (Figure 8D,F,H; Figure S15B,F). Similar results were found in the MAGE‐A3 (Figure 8J) and adoptive T‐cell therapy cohorts (Figure S15C,D). These data suggested that GBP score can be a promising predictor for immunotherapy response across cancers.

## 4 DISCUSSION

Previous studies have identified the roles of GBP family molecules in host‐pathogen infection and some cancers,^13^, ^15^, ^16^, ^30^ mainly focusing on their expression and prognostic values. But little is known about the roles in human liver disease. According to the existing research, GBP1 was repressed in hepatitis B virus‐infected patients,^31^ GBP2 expression levels were enhanced in patients with nonalcoholic fatty liver disease,^32^ and overexpression of GBP5 could induce liver injury and inflammation.^33^ Only two studies identified that low GBP1 expression was a risk factor for HCC recurrence and GBP5 had prognostic significance in HCC.^34^, ^35^ Therefore, the contribution of GBP molecules to HCC is largely undetermined. In this study, the genetic characterizations, prognostic values, and immunological effects of GBPs in HCC were preliminarily disclosed. We found almost all the GBP family members had markedly favorable prognostic values for patients with HCC in five independent cohorts, even though a few of them (such as GBP2) were not significant in some datasets. Thus we believed that the GBP family had the potential to affect the progression and predict the prognosis of HCC. Then, a GBPs‐score was constructed to integrate the values of GBP molecules by the PCA algorithm. Although lots of good prognostic models for HCC have been established, and the robust signature choosing genes from a wider pool may have stronger significance, we constructed the GBPs‐score aiming to provide a novel biomarker predicting prognosis, immunologic patterns, and immunotherapy response for HCC, as a complement to existing studies. Indeed, excellent performances of the GBPs‐score in these fields were demonstrated in our study.

Integrated evaluation on multiple independent cohorts of HCC displayed that GBPs‐score could act as an independent prognostic signature in HCC, and high GBPs‐score inferred better survival, which was verified by various methods (K‐M curve, univariate and multivariate Cox regression, and C‐index evaluation) to avoid calculation errors. We also found strong positive relationships between GBPs‐score and favorable clinicopathological features, and other HCC‐related favorable molecular signatures. These data collectively suggested GBPs were associated with a favorable prognosis in HCC patients.

As members of the IFN‐γ stimulated superfamily, GBPs are also induced by inflammatory cytokines such as TNF‐α, IL‐1α and IL‐1β in immune cells.^36^ The induction of GBPs by inflammatory cytokines indicates their contribution to inflammatory activities. Haque et al.^37^ reviewed the role of GBPs in the pathogenesis of autoimmune and inflammatory diseases such as inflammatory bowel disease, psoriasis, rheumatoid arthritis, etc. In our study, significant inflamed characteristics were found in the HCC samples with high GBPs‐score according to GSEA. In addition, the GSEA also showed a high GBPs‐score pointed to less tumor proliferation, and more immune‐related features (Figure 5A,B). Thus, we inferred GBPs may be involved in the anti‐tumor immunity in HCC. The primary step of an effective anti‐tumor response is the processing and presentation of antigens that mainly include HLA‐I molecules, which can be recognized by cytotoxic CD8^+^ T cells.^25^ Then the tumor cells can be killed by the cytotoxic CD8^+^ T cells. Besides, higher CYT was associated with more MHC‐I‐associated neoantigens and enhanced the immune response.^26^ Here, we found GBPs‐score was positively correlated with the APM gene expression and APM score, the CYT score, and the IFN‐γ signature in the samples with HCC. The cytokine IFN‐γ is essential in developing immune system cells with anti‐tumor activities.^38^, ^39^ A previous study demonstrated that an IFN‐γ‐driven TME with elevated GBP1 expression exhibited a superior prognostic effect in CRC.^40^ In addition, we found higher GBPs‐score was related to a higher immune score but lower tumor purity of HCC samples. Collectively, these data suggested that GBPs played an anti‐tumor role in HCC.

Activation and infiltration of immune cells correlate with a better prognosis of HCC, especially the cytotoxic CD8^+^ T cells with anti‐tumor activity.^41^ The heightened effector functions of cytotoxic CD8^+^ T cells and tumor clearance require the support of Th1 cells, which are the most important anti‐tumor helper T cells.^42^, ^43^ Here, we revealed high GBPs‐score was highly correlated with an immune‐hot TME that infiltrated abundant immune cells, including not only the cells with anti‐tumor effects but also some immunosuppression‐related cells (such as Tregs). Even so, the anti‐tumor immune cells (especially CD8^+^ T and Th1 cells) were more significant than the others, suggesting the improvement of the GBPs‐score was more related to the activation of anti‐tumor immunity. In terms of individual GBP genes, GBP1‐5 had stronger correlations with the infiltration of the anti‐tumor cells (Figure 6B,D) and the immune‐hot TME (Figure 5H) than GBP6/7.^12^ Given the above results were based on bioinformatics prediction, single‐cell sequencing data and IHC assays of a TMA cohort were supplemented. We confirmed that GBP molecules were more active in CD8^+^ T cells than in Tregs, and only GBP6/7 was not or rarely expressed in HCC immune cells. These data suggested GBP1‐5 rather than GBP6/7 was highly correlated with enriched CD8^+^ T cells. Furthermore, the positive correlation of GBP1‐5 expression and infiltration of CD8^+^ T cells was confirmed in our TMA cohort. Also, the TMA analysis verified that high GBP1‐5 was associated with favorable features of HCC patients, consistent with the effects of GBPs‐score. In sum up, a higher GBPs‐score in HCC infers to more abundant immune infiltrations, in which the anti‐tumor cells, such as CD8^+^ T cells, play a dominant role. Thus the patients with high GBPs‐scores may have more activated anti‐tumor immunity, and thereby these patients were closely associated with better prognoses.

As mentioned earlier, tumors can be divided into “hot” and “cold” tumors, and immune “hot” tumors are responsive to cancer immunotherapy.^8^ In our study, the HCC samples with high GBPs‐score showed more active IFN‐γ response, abundant CTL infiltration, and higher APM and CYT levels. The APM is a fundamental determinant of tumor immunogenicity,^44^ which is positively affected by CYT,^44^ and antigen presentation defects could contribute to ICI‐response failure.^45^ Therefore, we suggested that the immune microenvironment of HCC patients with high GBPs scores tended to be “hot” and such patients were more likely to benefit from immunotherapy. ICB directly stimulates the activation of CTL to achieve anti‐tumor effects by blocking inhibitory signaling.^46^ The crucial immune checkpoints include PD‐1, PD‐L1, and CTLA4. Here, GBPs‐score was positively related to the expressions of these crucial checkpoints and ICB response‐related signatures. Also, the SubMap analysis suggested that patients with HCC in the high GBPs‐score groups had a higher likelihood of response to anti‐PD1 treatment. Besides, higher GBPs‐score inferred higher response rates and better prognosis across several immunotherapy‐related cohorts with different cancers. In summary, we believe that the GBPs‐score is a promising indicator to predict the immunotherapy response. As for individual GBP genes, GBP1‐5 was more dominant than GBP6‐7 in HCC TME in our study. The TMA analysis showed GBP1‐5 was significantly correlated with PD‐L1 and CD8, further suggesting higher GBP1‐5 expression was more likely to respond to anti‐PD1 treatment. Likewise, our previous study^17^ revealed GBP2 could serve as a therapeutic target for ICB treatment of sensitization in microsatellite stability CRC. Collectively, GBP molecules may be responsive to immunotherapy response, and GBP1‐5 can be potential immunotherapy targets in HCC.

Our study has some novelty and strengths. Firstly, we are the first to comprehensively explore the role of GBP family molecules in HCC. Their genetic characteristics, prognostic values, and immune roles in HCC were analyzed integratedly and separately. Secondly, we used the PCA method to construct the GBPs‐score based on GBP1‐7, although only GBP1‐5 showed stronger correlations in determining immune status and prognosis in HCC. This was because the PCA algorithm focuses the score on the set with the most well‐correlated (or anti‐correlated) genes in the set, while down‐weights contributions from genes with weak correlations. Therefore, the GBPs‐score was not a simple addition of gene effects, but rather a full integration of gene values. Thirdly, the GBP family signature (GBPs‐score) showed excellent performances, which were validated in multiple independent HCC cohorts. Additionally, its predictive ability in immunotherapy can be extended to other cancers. These data indicated the GBPs‐score can be a promising prognostic and immunologic biomarker for HCC.

Some limitations of this study need to be noted. Firstly, although a TMA cohort with 94 human specimens was included, our study was mainly carried out using bioinformatics methods through a public database. Further experiments and clinical studies are required to verify the role of GBPs in the future. Secondly, although the excellent performance of the GBPs‐score was validated in multiple cohorts, we should explore in‐depth mechanisms of the relationships between GBPs in future researches. Thirdly, despite the excellent performance of GBPs‐score was shown, adding some non‐GBP genes with stronger statistical significance into the signature may further improve the diagnostic performance. Fourthly, due to lacking of immunotherapy data on HCC, the enrolled immunotherapy groups for analysis are non‐HCC, which cannot directly reflect the predictive value of GBPs‐score in HCC immunotherapy. To compensate for this limitation, we conducted the Submap analyses in HCC cohorts and PD‐L1 IHC assays in the TMA cohort, which could confirmed the correlation between GBPs and the response to anti‐PD1 treatment. Fifthly, we focused on the role of GBPs in tumor immune response, while the exploration of other biological effects of GBPs was limited.

## 5 CONCLUSION

This integrated analysis revealed the genetic characterizations and immunologic roles of GBP family genes in HCC. The GBPs‐score was able to quantify integrative values of GBPs, predict the prognosis of patients with HCC, identify distinct immunologic patterns of HCC microenvironment, and help guide more effective immunotherapy strategies in cancers.

## AUTHOR CONTRIBUTIONS

**Yumei Ning:** Data curation (equal); formal analysis (lead); methodology (equal); visualization (equal); writing – original draft (lead). **Shilin Fang:** Data curation (lead); methodology (equal); writing – original draft (equal). **Jun Fang:** Conceptualization (equal); methodology (equal); supervision (lead). **Kun Lin:** Data curation (supporting); formal analysis (supporting). **Haihang Nie:** Visualization (supporting); writing – original draft (supporting). **Peiling Xiong:** Writing – original draft (supporting). **Peishan Qiu:** Methodology (supporting); writing – review and editing (supporting). **Qiu Zhao:** Funding acquisition (supporting); project administration (lead); writing – review and editing (equal). **Haizhou Wang:** Formal analysis (equal); methodology (lead); writing – review and editing (lead). **Fan Wang:** Conceptualization (equal); funding acquisition (lead); writing – review and editing (equal).

## CONFLICT OF INTEREST STATEMENT

The authors declare no conflict of interest.

## ETHICS STATEMENT

The use of human specimens was approved by Ethics Committee of Zhongnan Hospital of Wuhan University (No. 2020011). All methods were carried out by the relevant guidelines under the ethical approval and consent to participate section.

## Supporting information


Figure S1.
Figure S2.
Figure S3.
Figure S4.
Figure S5.
Figure S6.
Figure S7.
Figure S8.
Figure S9.
Figure S10.
Figure S11.
Figure S12.
Figure S13.
Figure S14.
Figure S15.

Data S1.

Data S2.

Table S5.
Table S6.
Table S7.
Table S8.
Table S10.
Table S11.
Table S12.

## Data Availability

This study involved publicly available datasets, which can be found at https://xenabrowser.net/datapages/, https://www.ncbi.nlm.nih.gov/geo/, and https://icgc.org/. The supplementary materials of this article are available online. The R codes used in this study can be available on request reasonably from the corresponding author.
