# Supplementary material for: Guanylate‐binding proteins signature predicts favorable prognosis, immune‐hot microenvironment, and immunotherapy response in hepatocellular carcinoma
Source: Cancer Med. 2023 Aug 7;12(16):17504–21. doi: 10.1002/cam4.6347 (PMC10501289; doi:10.1002/cam4.6347)
Supplement: Supplementary file 1 — Figure S1. Figure S2. Figure S3. Figure S4. Figure S5. Figure S6. Figure S7. Figure S8. Figure S9. Figure S10. Figure S11. Figure S12. Figure S13. Figure S14. Figure S15. [file CAM4-12-17504-s003.docx]

**Supplementary figures**

**
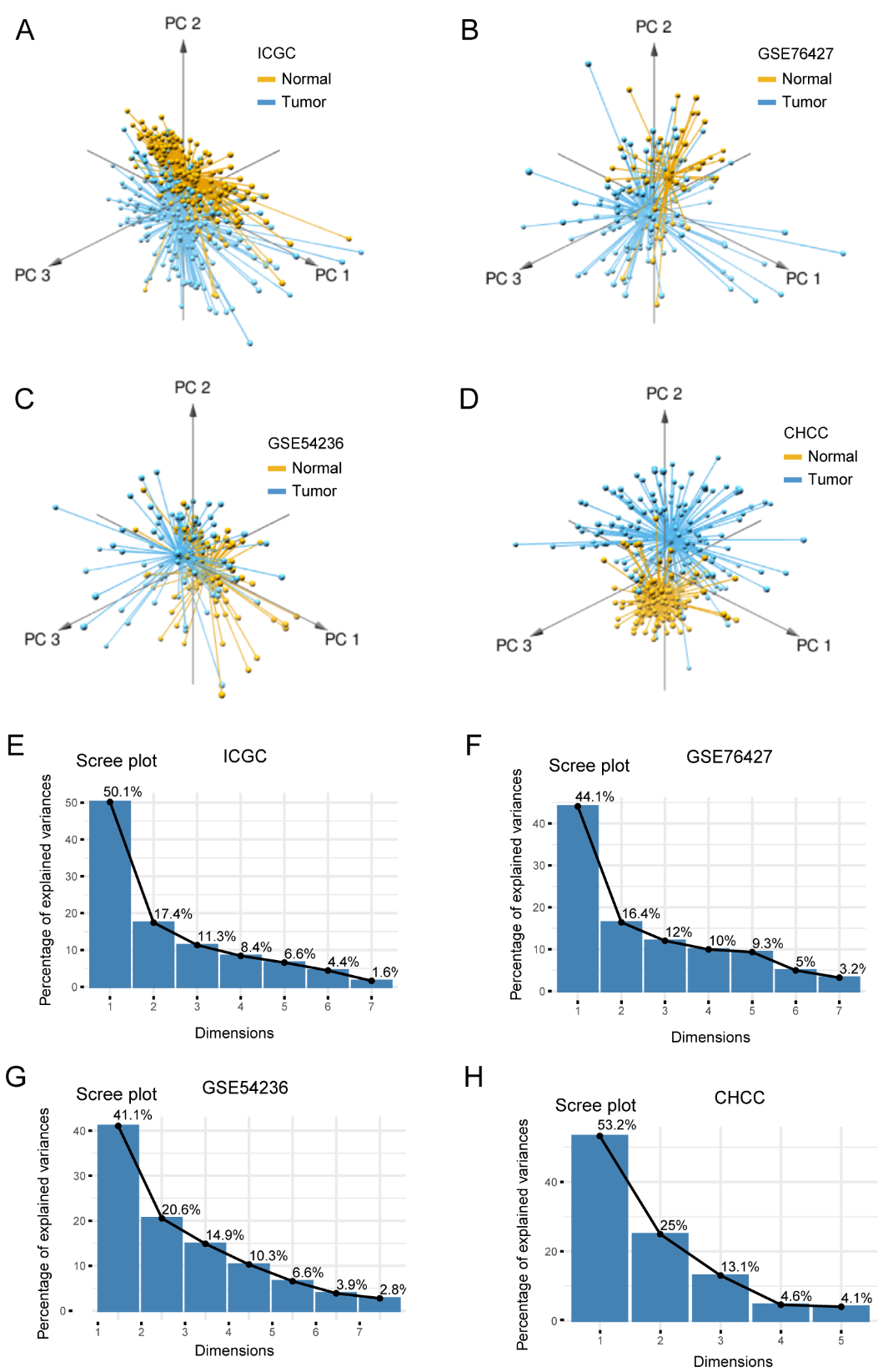
**

**Figure S1 (A-D)** Principal component analysis (PCA) for the expression profiles of seven GBPs could distinguish tumors from normal samples in the ICGC **(A)**, GSE76427 **(B)** and GSE54236 **(C)**, and CHCC **(D)** cohorts. **(E-H)** Cumulative variance plots of the PCA in the ICGC **(E)**, GSE76427 **(F)** and GSE54236 **(G)**, and CHCC **(H)** cohorts. GBP3 and GBP6 expression data were missing in the CHCC cohort.


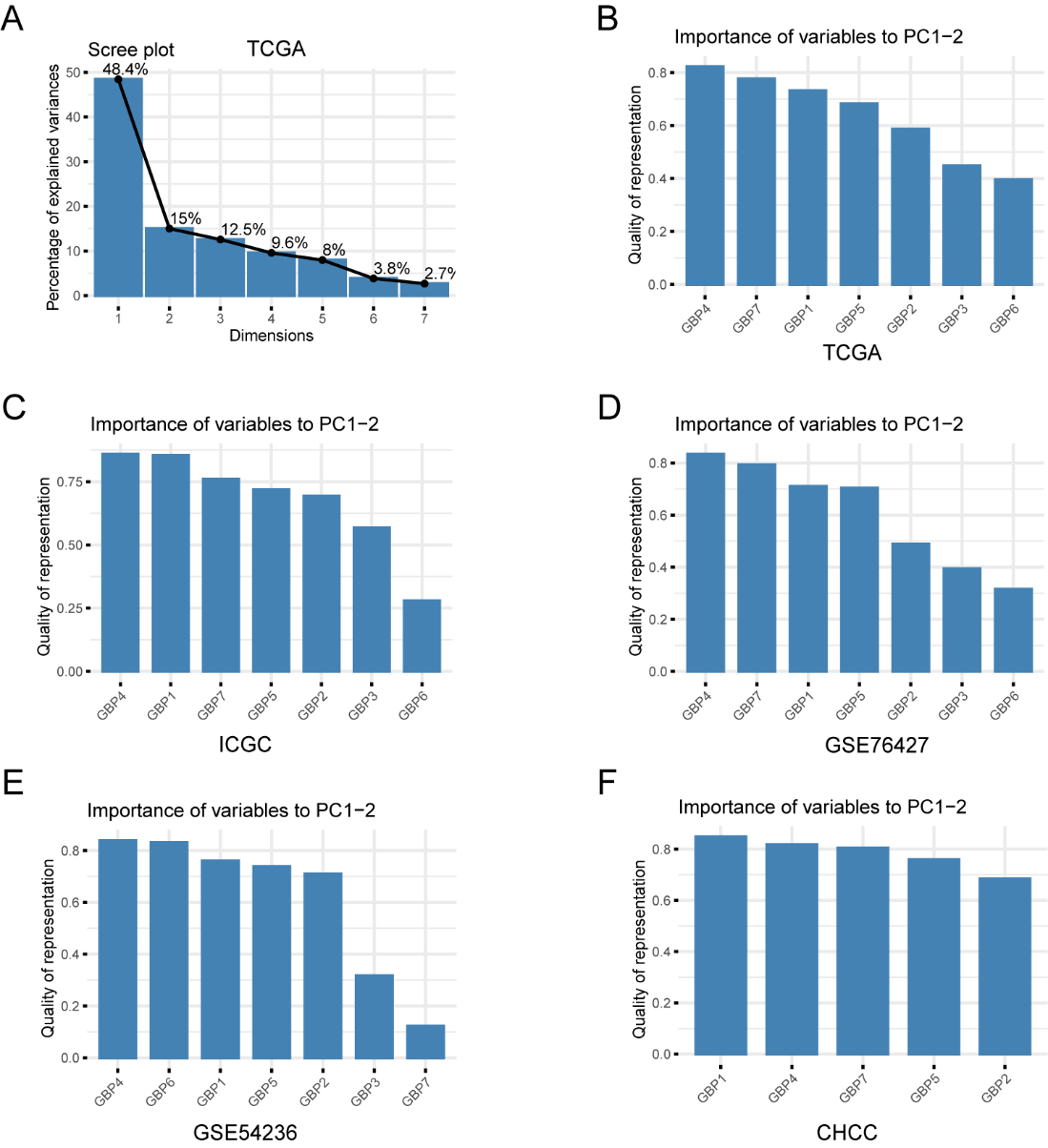


**Figure S2** **(A)** Cumulative variance plots of the PCA in the TCGA cohort. **(B-F)** The importance of each GBP molecule to PC1 and PC2 in the PCA of five HCC datasets. GBP3 and GBP6 expression data were missing in the CHCC cohort.


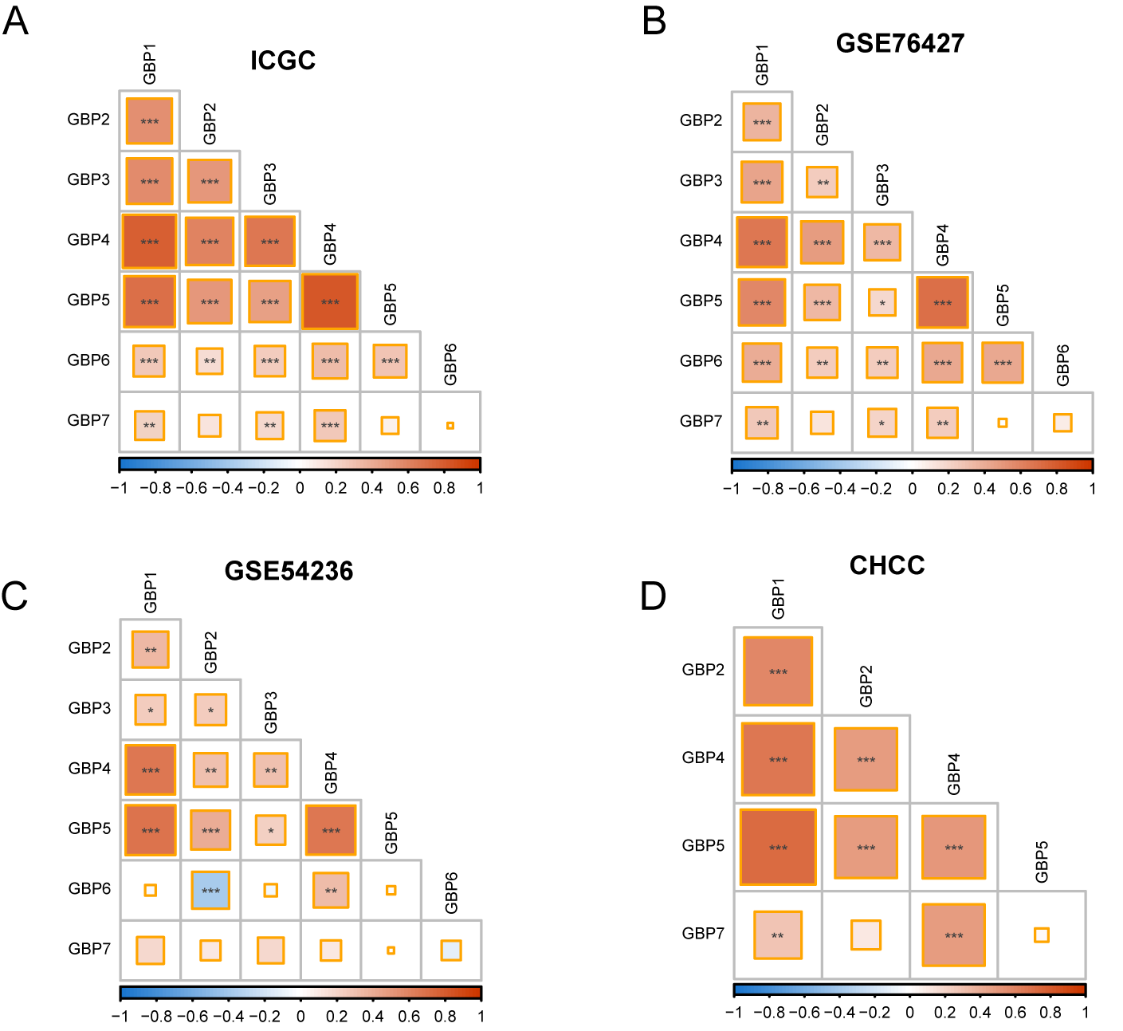


**Figure S3.** The correlation of GBP family molecules in the ICGC **(A)**, GSE76427 **(B)** and GSE54236 **(C)**, and CHCC **(D)** cohorts. GBP3 and GBP6 expression data were missing in the CHCC cohort.


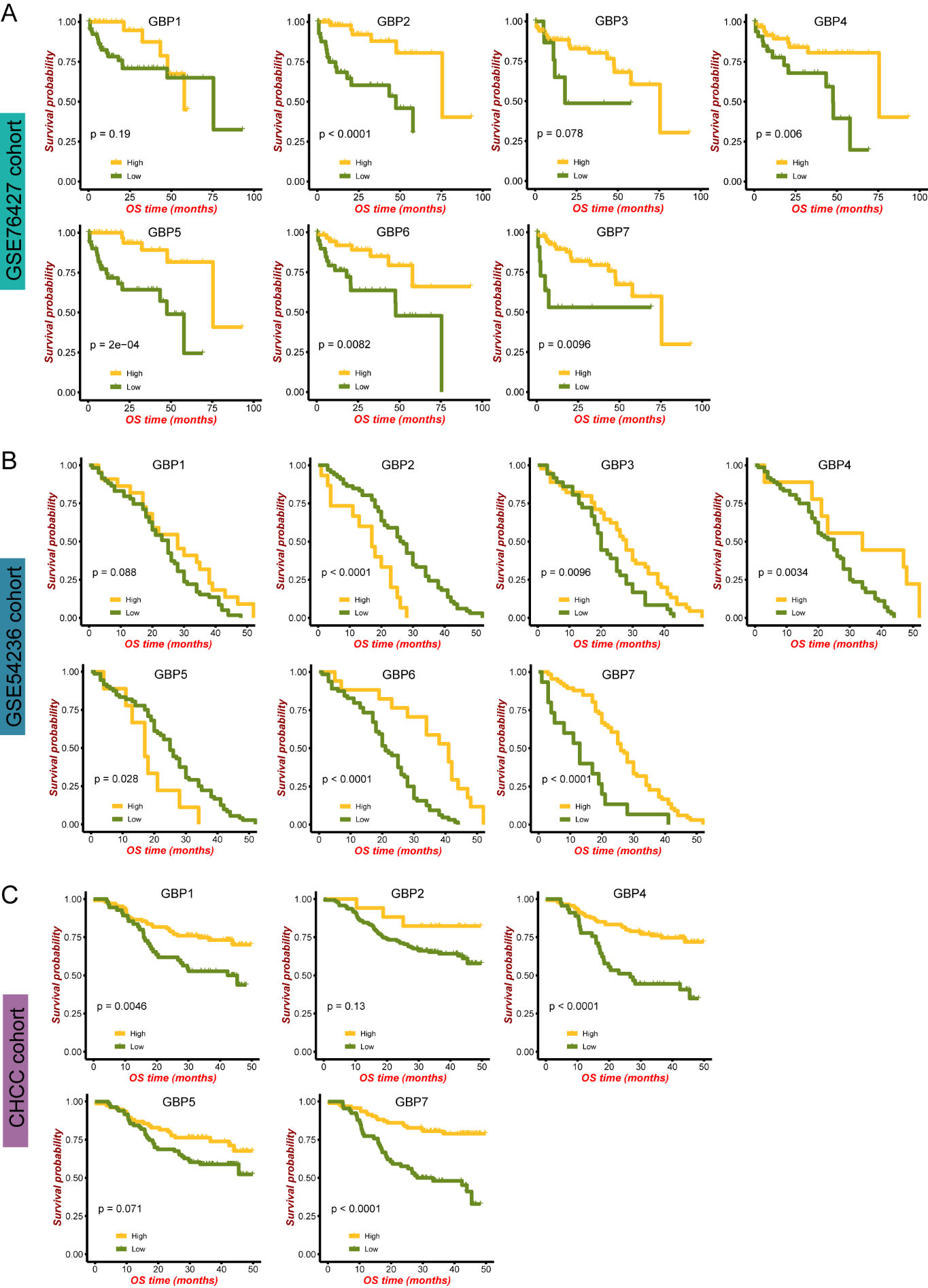


**Figure S4**. Correlations between GBP1-7 expression and prognosis of HCC patients in the GSE76427 **(A)** and GSE54236 **(B)**, and CHCC **(C)** cohorts. GBP3 and GBP6 expression data were missing in the CHCC cohort.

**
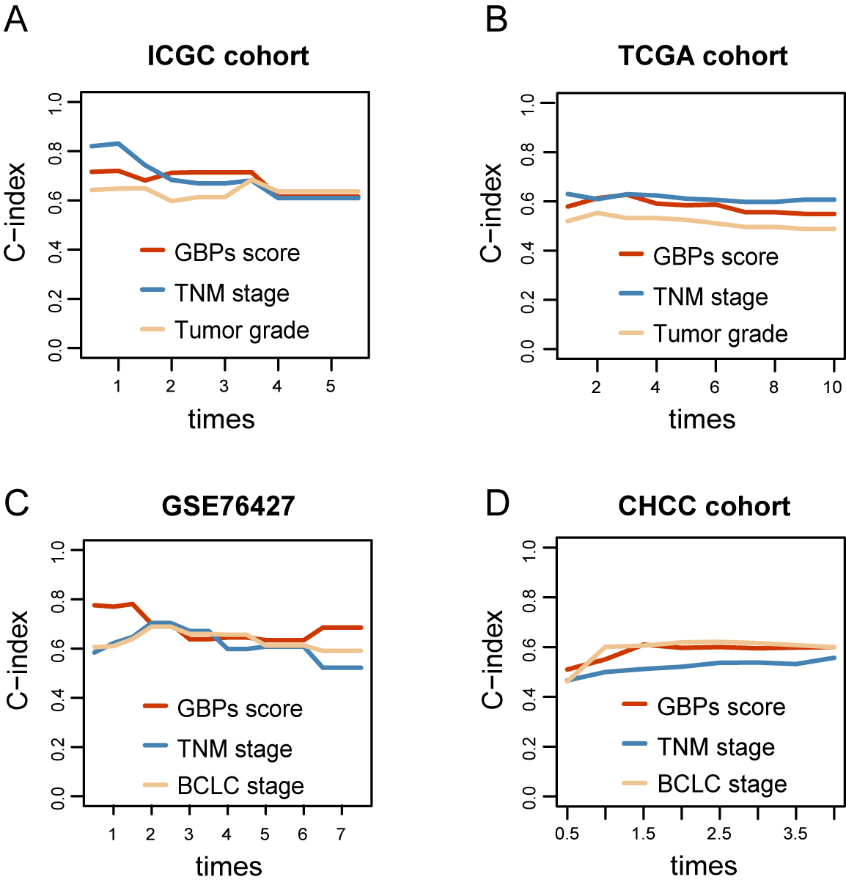
**

**Figure S5** Time-dependent C-indexes of GBPs-score and clinical variates in ICGC **(A)**, TCGA **(B)**, CHCC **(C)**, and GSE76427 **(D)** cohorts.


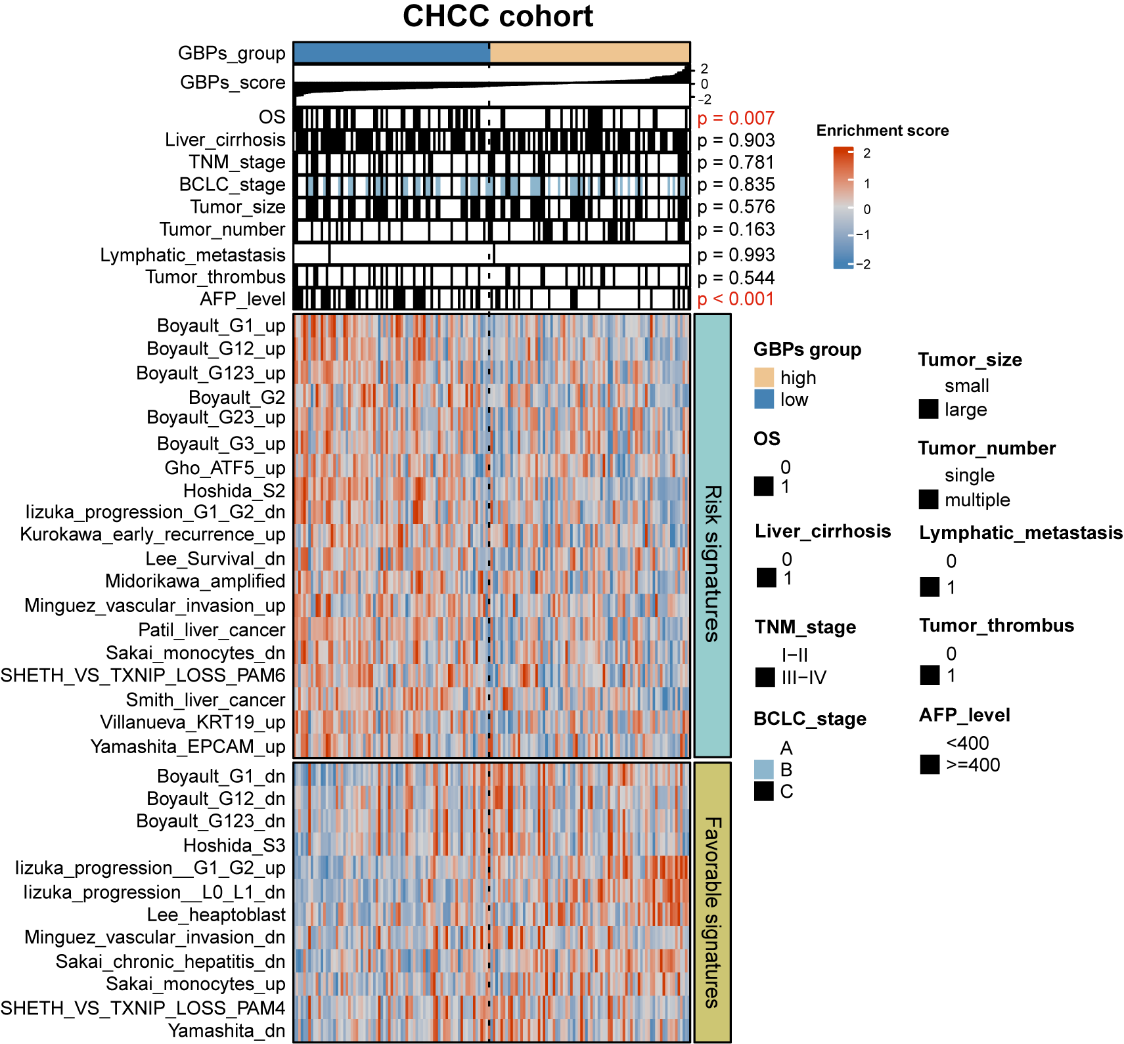


**Figure S6** Relationships between GBPs-score and clinical features and other HCC-related molecular signatures in the CHCC cohort.


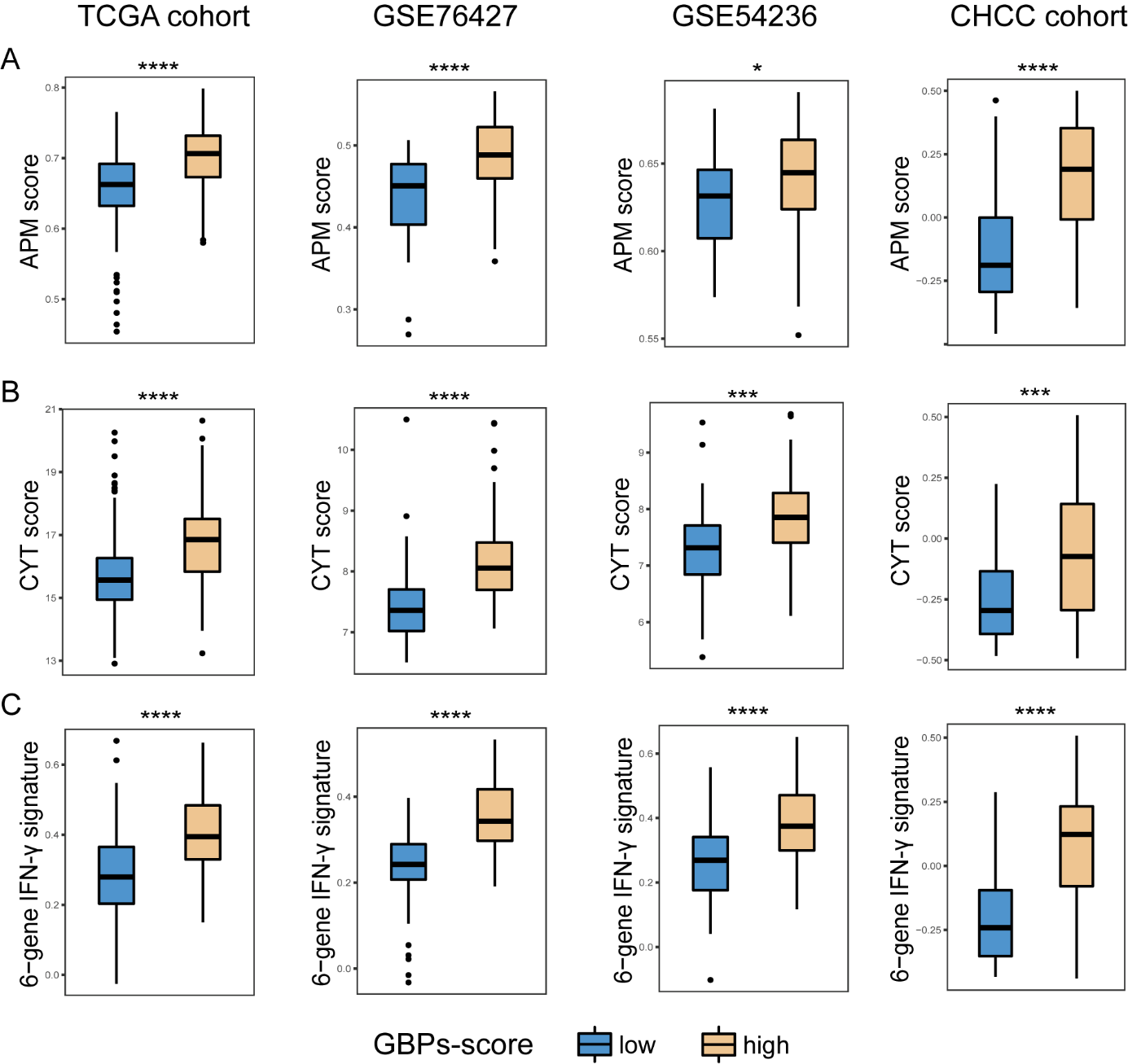


**Figure S7** Differences of APM score **(A)**, CYT score **(B)**, and 6-gene IFN-γ signature **(C)** in GBPs-score subgroups.


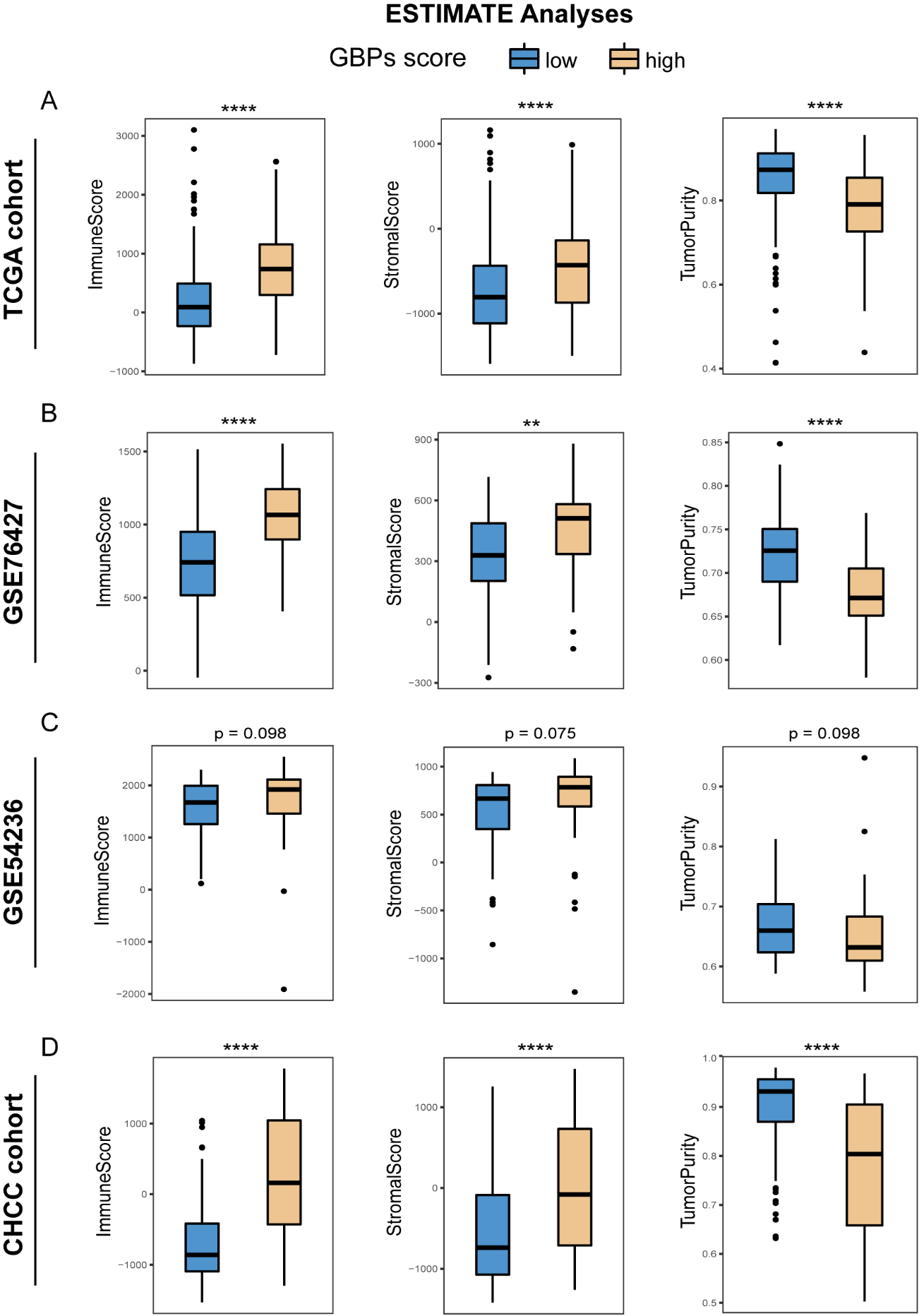


**Figure S8** ESTIMATE analyses showed the differences of immune score, stromal score, and tumor purity in GBPs-score subgroups in TCGA **(A)**, GSE76427 **(B)**, GSE54236 **(C)**, and CHCC cohorts **(D)**.


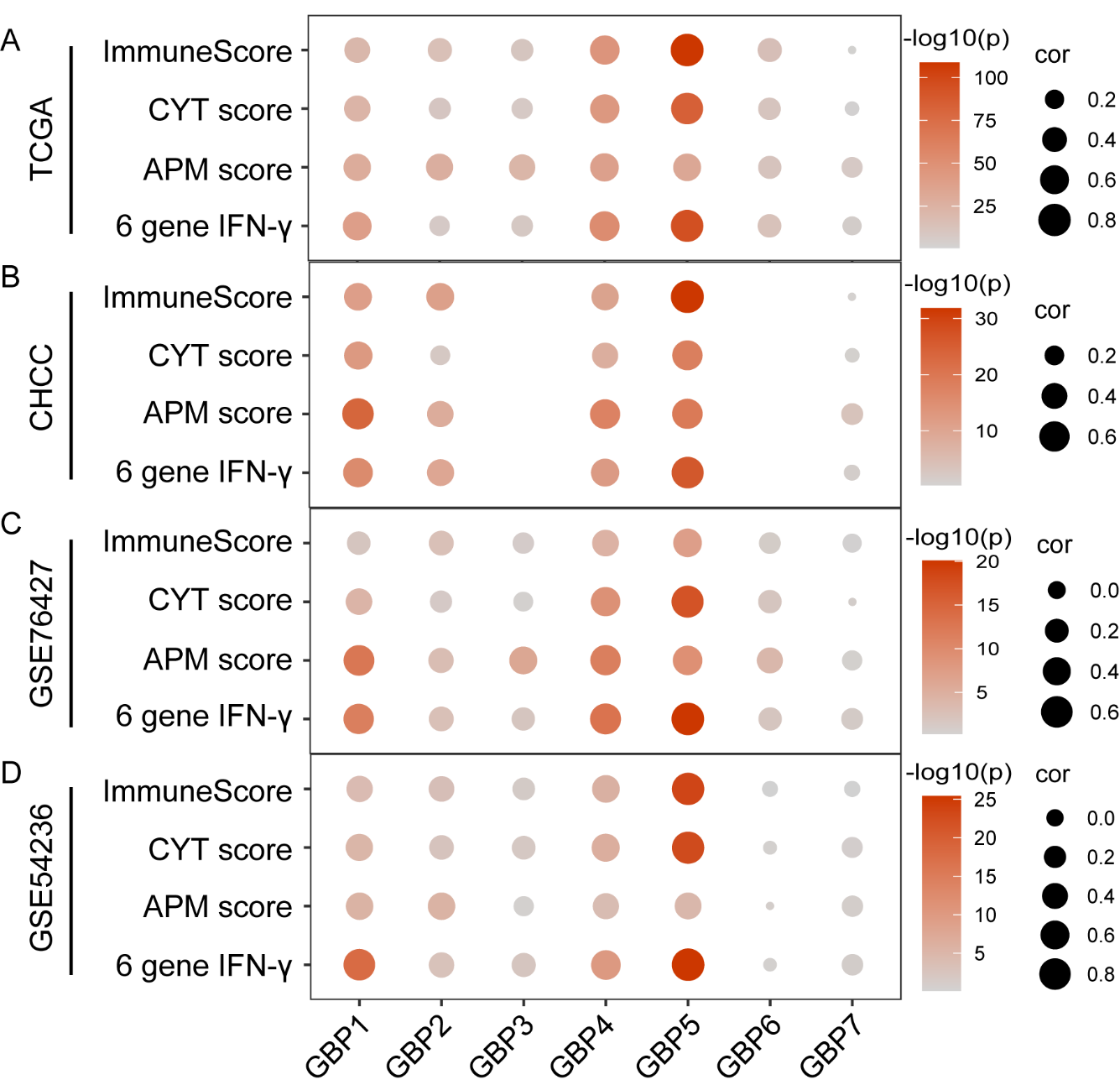


**Figure S9** Bubble plots show the correlations between GBPs-score and immune score, CTY score, APM score, and 6-gene IFN-γ signature in TCGA **(A)**, CHCC **(B)**, GSE76427 **(C)**, and GSE54236 **(D)** cohorts.


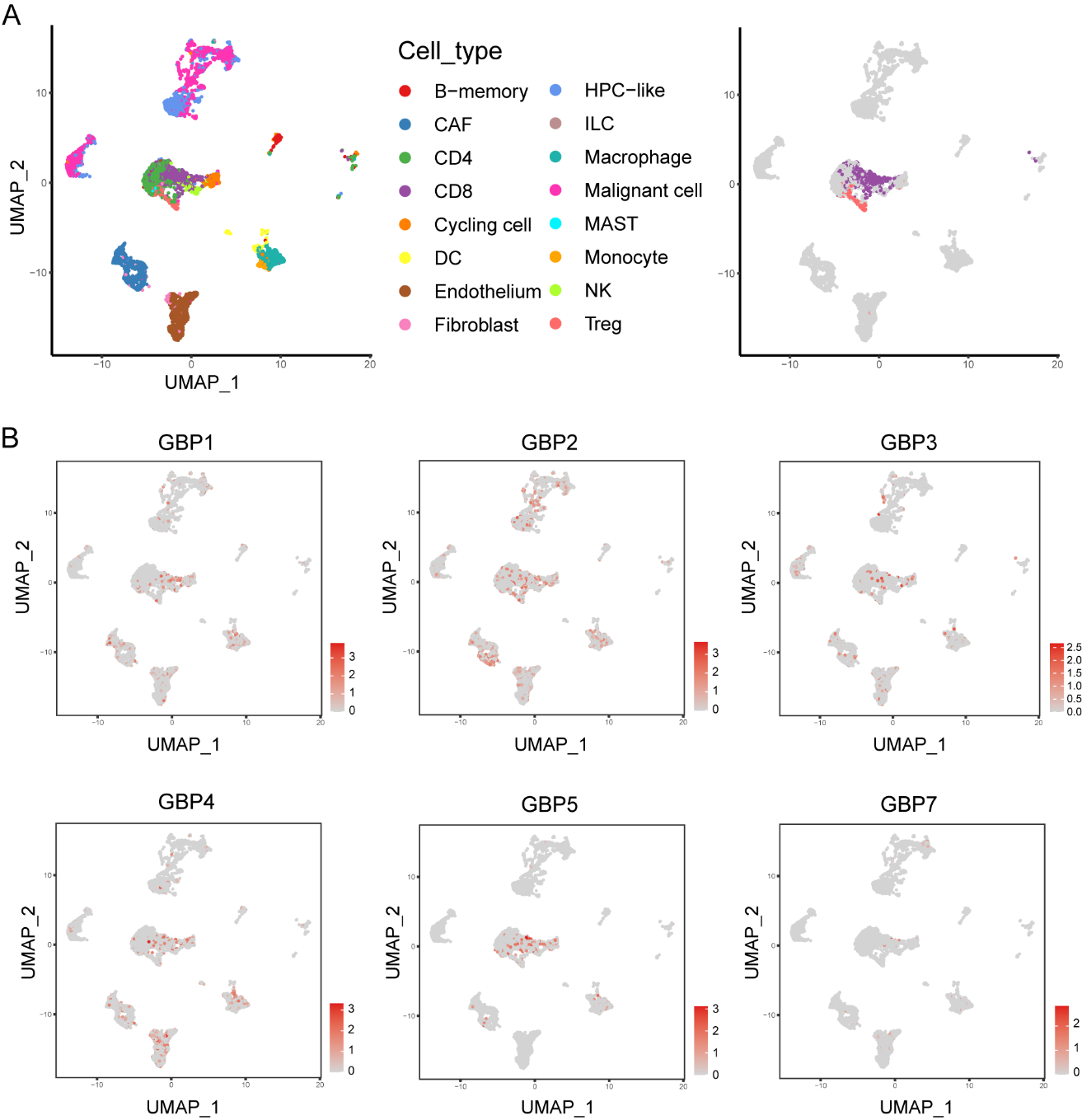


**Figure S10** Single-cell RNA sequencing analysis of the GBP gene expression in HCC immune cells. The analysis was performed in http://sctime.sklehabc.com/. **(A)** UMAP plot showing 16 major cell types using the single-cell RNA sequencing data from 19 cases of HCC in GSE125449. **(B)** Expressions of GBP family gens on UMAP clustering, cells were colored according to the corresponding expression of molecules.


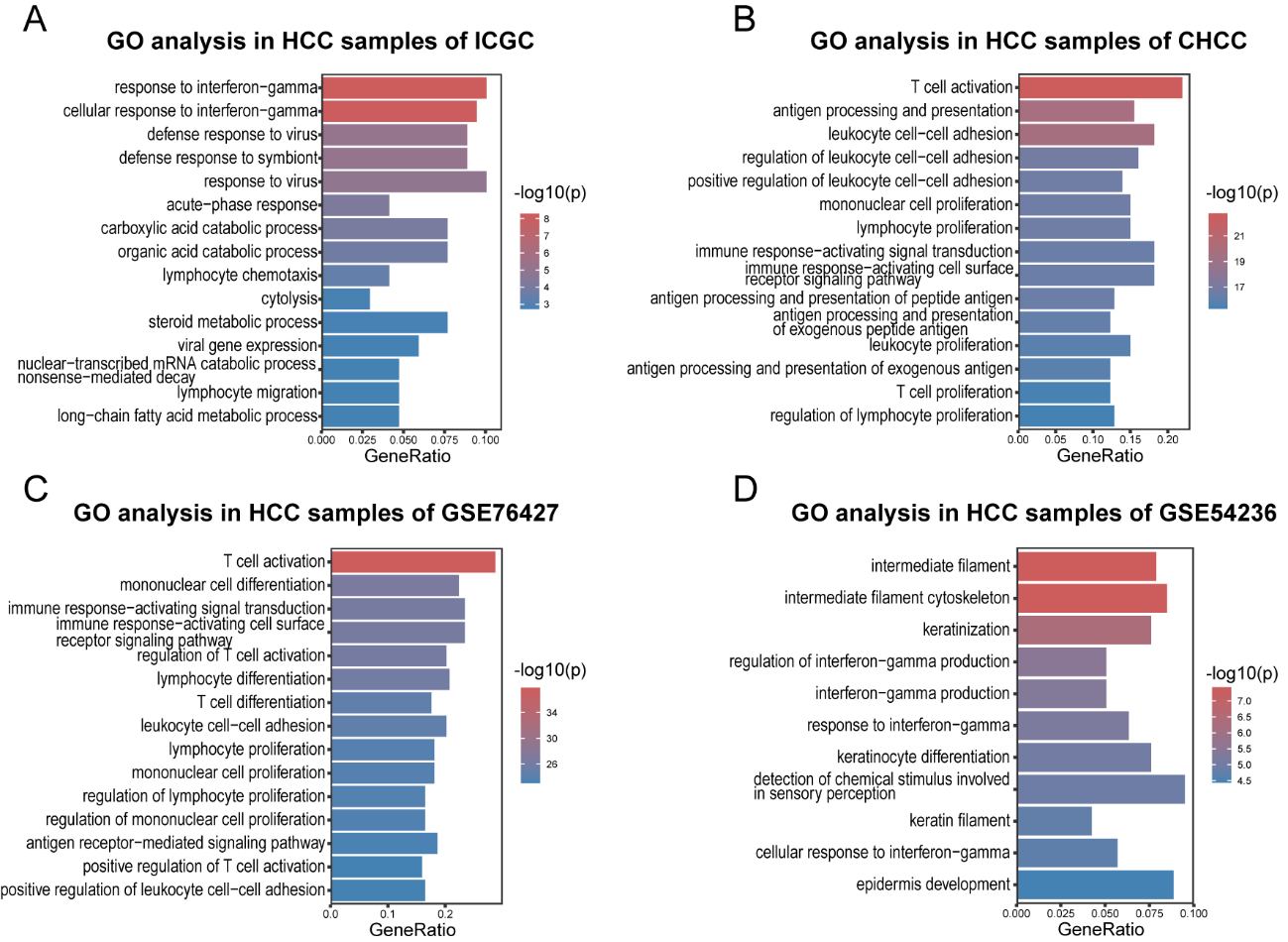


**Figure S11** GO analysis based on the top 200 differential expressed genes that were screened between high vs low GBPs-score in the ICGC **(A)**, CHCC **(B),** GSE76427 **(C)**, and GSE54236 **(D)** cohorts.


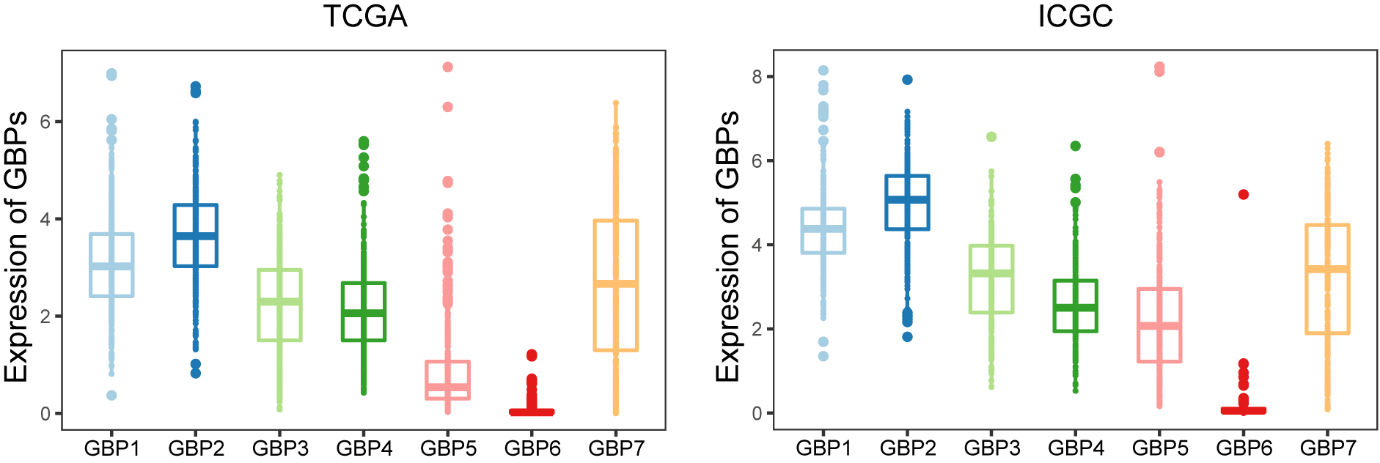


**Figure S12** Expression analysis of GBP molecules in HCC tissues using RNA-sequencing data.


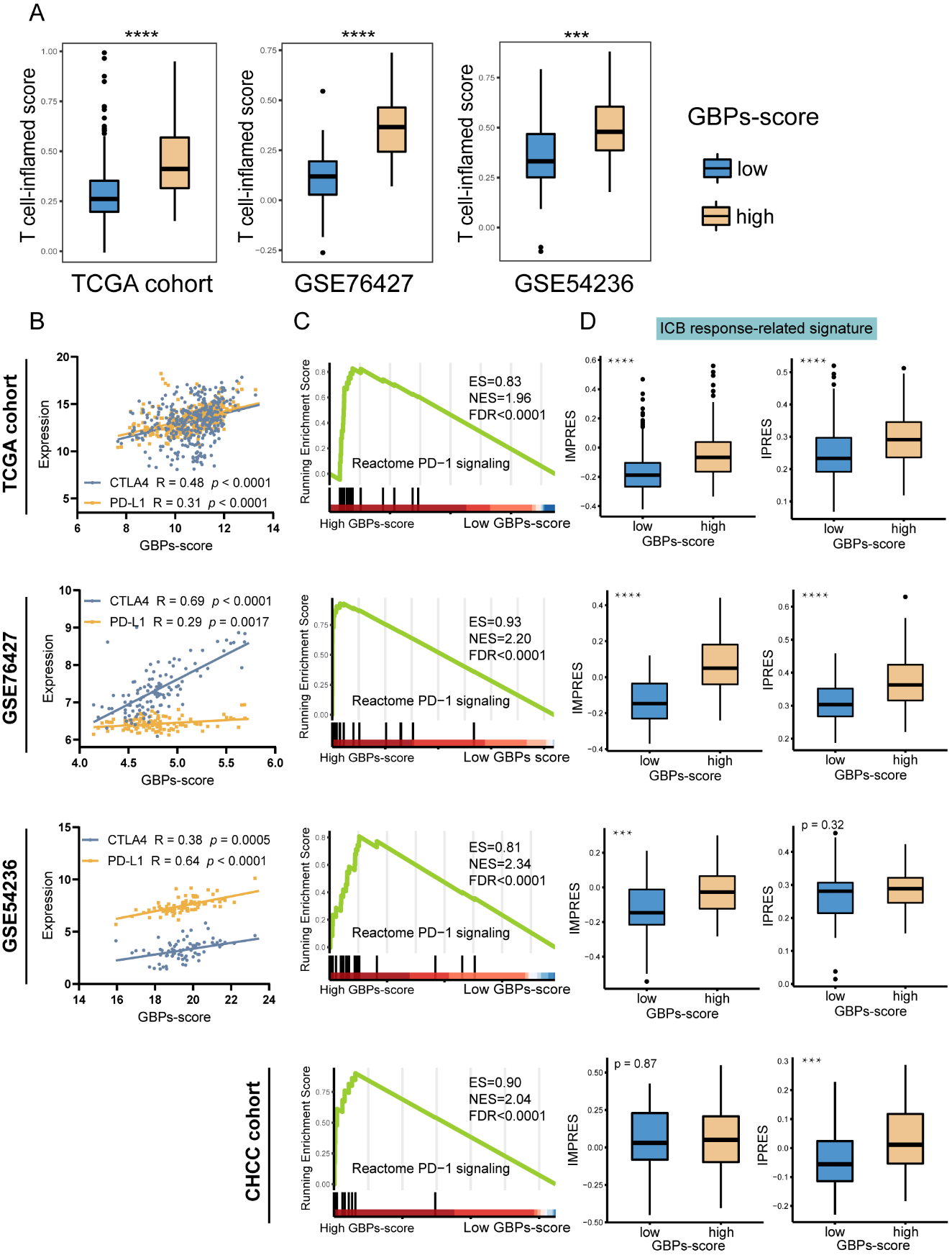


**Figure S13 (A)** T cell-inflamed GEP were higher in high GBPs-score groups according TCGA, GSE76427 and GSE54236 cohorts. **(B)** Correlation of GBPs-score and expression of PD-L1 and CTLA4. **(C)** GSEA plots showed reactome PD-1 signaling had positive correlations with higher GBPs-scores. **(D)** Box plots showed the levels of ICB response-related signatures between GBPs-score subgroups.


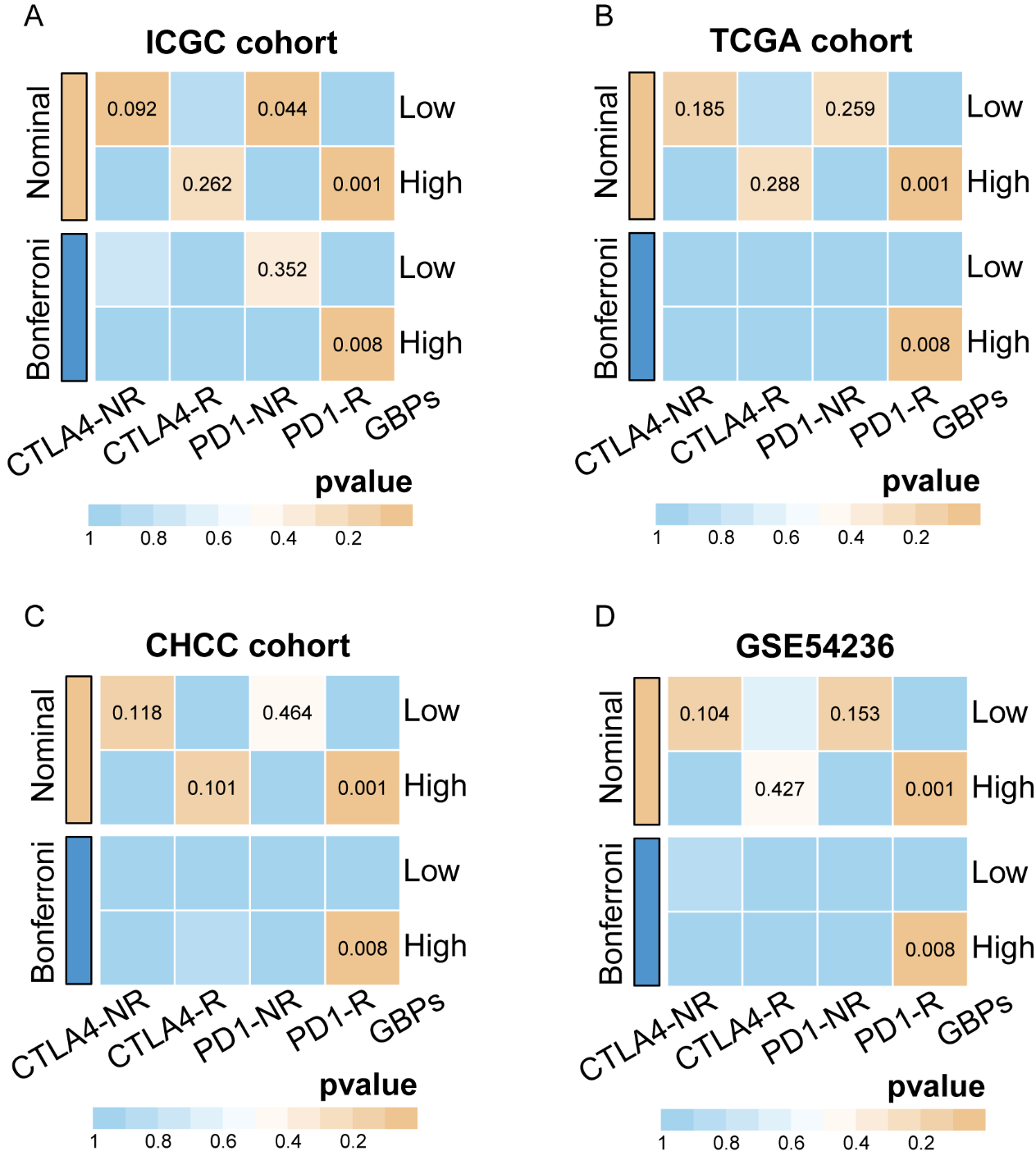


**Figure S14** SubMap analysis revealed that high GBPs-score group exhibited a high likelihood of response to anti-PD1 in the HCC samples of ICGC **(A),** TCGA **(B),** CHCC **(C),** GSE54236 **(D)**.

**
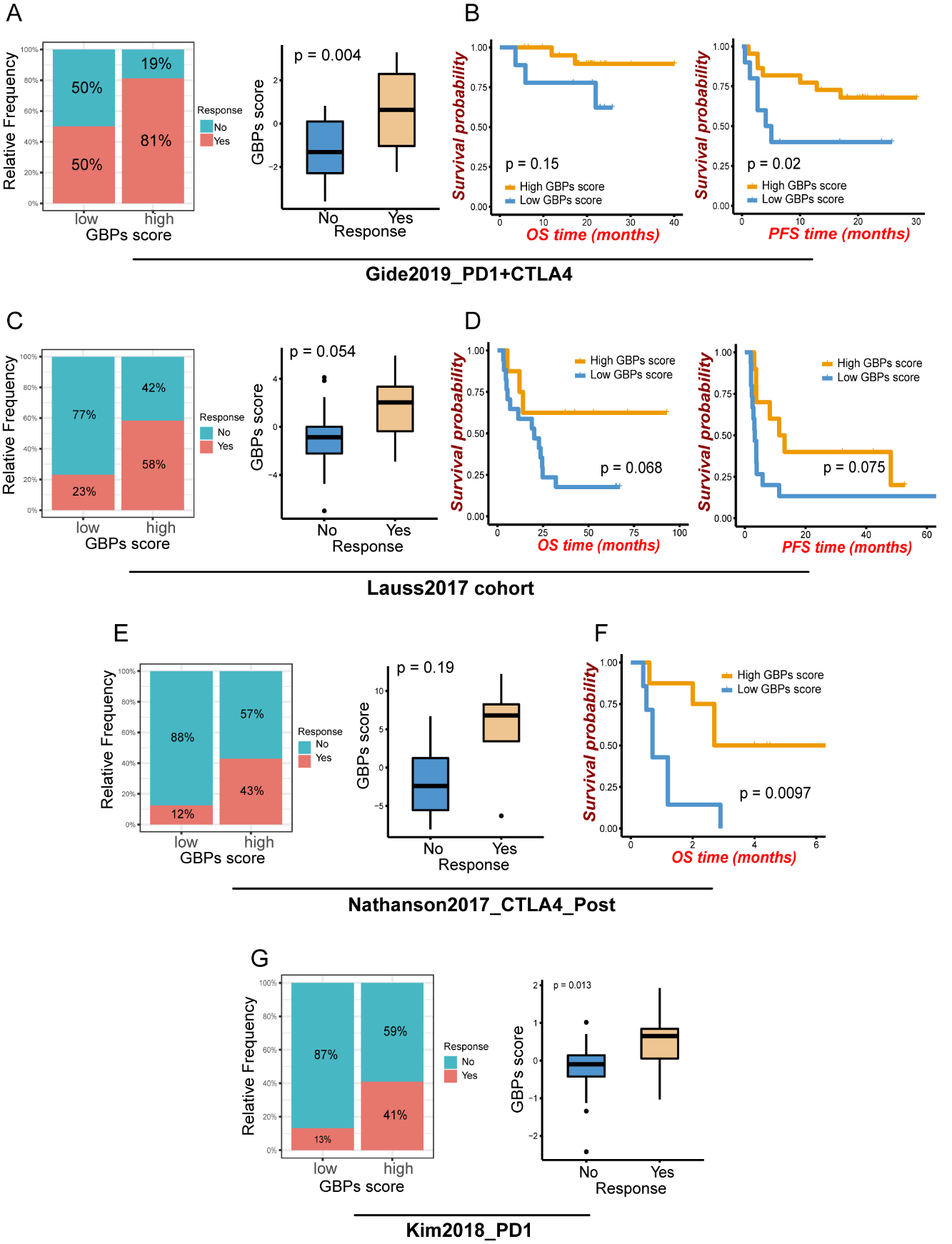
**

**Figure S15** Correlation of GBPs-score and immunotherapy response in Gide2019 (melanoma) **(A)**, Lauss2017 (melanoma) **(C)**, Nathanson2017 (melanoma) **(E)** and Kim2018 (gastric cancer) cohorts **(G)** cohorts. K-M curves showed the relationships between GBPs-score and survival outcomes of patients in Gide2019 (melanoma) **(N)**, Lauss2017 (melanoma) **(D)** and Nathanson2017 (melanoma) **(F)** cohorts**.**
