# Supplementary material for: Guanylate‐binding proteins signature predicts favorable prognosis, immune‐hot microenvironment, and immunotherapy response in hepatocellular carcinoma
Source: Cancer Med. 2023 Aug 7;12(16):17504–21. doi: 10.1002/cam4.6347 (PMC10501289; doi:10.1002/cam4.6347)
Supplement: Supplementary file 2 — Data S1. [file CAM4-12-17504-s002.docx]

**Supplemental methods**

- 1. **Immunotherapy cohorts**

In addition, to test the predictive value of immunotherapy responses of GBPs-score, eight immunotherapy cohorts were recruited: IMvigor210 cohort (bladder cancer, anti-PD-L1) were obtained from <http://research-pub.Gene.com/imvigor210corebiologies/> ^1^. GSE35640 (melanoma and non-small-cell lung cancer, MAGE-A3) and GSE126044 (non-small-cell lung cancer, anti-PD-1) were downloaded from the GEO database. Another five immunotherapy cohorts with a detailed RNA expression matrix and clinical information were gathered from the TIDE website (<http://tide.dfci.harvard.edu/download/>) ^2^.

- 1. **Evaluation of prognostic values of GBP molecules and GBPs-score for patients with HCC**

First, Kaplan-Meier (K-M) analysis was performed to evaluate the prognostic values of GBP molecules and GBPs-score in HCC patients using the "survival" R package. For individual GBP molecules, we aimed to investigate their prognostic values preliminarily, so the "survminer" R package and the “surv_cutpoint” function was used to investigate the optimal cutoff. For the GBPs-score, we aimed to determine its diagnostic performance and establish a stable prognostic model, so we selected the median of the GBPs-scores as the cutoff value. Also, the HCC samples from each cohort were divided into high and low GBPs-score groups by the median GBPs-score, and univariate Cox was performed between GBPs-score subgroups to further confirmed the prognostic value of the GBPs-score. And multivariate Cox regression analysis was conducted to adjust the influences of other factors to determine the prognostic value of GBPs-score. Finally, the concordance index (C-index) was calculated to investigate the prediction accuracy of GBPs-score and other clinical variables.

- 1. **Single-sample gene set enrichment analysis (ssGSEA) of other HCC-related molecular classification signatures**

To compare the GBPs-score with other HCC-related molecular gene signatures, we collected 75 gene signatures associated with HCC prognosis from the Molecular Signatures Database (MSigDB, http://www.gsea-msigdb.org/gsea/index.jsp) and previous research **(Table S20)** ^3^. The “GSVA” R package and ssGSEA algorithm were used to calculated the enrichment scores of these signatures ^4^. Complex heatmaps were built to show the correlations between these HCC-related molecular classifications and GBPs-score subgroups using the “ComplexHeatmap” R package.

- 1. **Gene set enrichment analysis (GSEA) and Gene Ontology (GO) functional annotation**

Based on the gene sets (“h.all.v7.5.symbols” and “c2.cp.kegg.v7.5.symbols”) retrieved from MSigDB, GSEA was performed to analyze the significant signaling pathways between GBPs-score subgroups. Additionally, GO functional enrichment analysis for the top 200 differentially expressed genes (DEGs) that were screened between high and low GBPs-score groups (separated by the median GBPs-score) by “limma” was performed using the “clusterProfiler” R package.

- 1. **Analyses of immune-related features**

The T cell-inflamed score composed of 18 inflammatory genes ^5^, and previously reported immune-related gene signatures were calculated with the transcriptome profiling data using the ssGSEA algorithm (R package “GSVA”). The cytolytic activity (CYT) was calculated using previously described computational methods ^6^. The "Estimation of Stromal and Immune cells in Malignant Tumors using Expression data" (ESTIMATE) algorithm was used to assess the immune score, stromal score and tumor purity for the patients with HCC ^7^.

The relative abundance of 28 immune cell infiltration in each HCC sample was quantified via ssGSEA **(Table S21)**. Other immune infiltration related signatures were collected from previous studies, and the enrichment score of each sample was also calculated by ssGSEA algorithm **(Table S22)**. The cancer-immunity cycle that describes the body activates the anti-tumor immune response to kill tumor cells included seven critical steps: release and presentation of cancer cell antigens (Steps 1 and 2), priming and activation of anti-cancer immune (Step3), immune cell trafficking (Step 4), infiltrate the tumor bed (Step 5), recognition of cancers by T cell (Step 6), and killing of cancer cells (Step 7) ^8^. The ssGSEA was used to assess the relative activities of the cancer-immunity cycle as previously described ^9^. The “limma” R package was used to screen the differentially immunity activities in the tumors between high and low GBPs-score groups (separated by the median GBPs-score). The other public available immune-related signatures were summarized in **Table S22**. The single-cell transcriptome analysis for liver cancer patients (10X Genomics) of GSE125449 was performed at http://sctime.sklehabc.com/ ^10^.

The subclass mapping (SubMap) method (GenePattern module ‘SubMap’) (https://cloud.genepattern.org) was used to evaluate the correspondence of GBPs-score subgroups and the patients with different responses from a melanoma dataset including anti-PD-1 and anti-CTLA4 therapy ^11,12^.

**Reference**

1. Mariathasan S, Turley SJ, Nickles D, et al. TGFβ attenuates tumour response to PD-L1 blockade by contributing to exclusion of T cells. *Nature.* 2018;554(7693):544-548. doi:10.1038/nature25501

2. Jiang P, Gu S, Pan D, et al. Signatures of T cell dysfunction and exclusion predict cancer immunotherapy response. *Nature medicine.* 2018;24(10):1550-1558. doi:10.1038/s41591-018-0136-1

3. Chen S, Gao Y, Wang Y, Daemen T. The combined signatures of hypoxia and cellular landscape provides a prognostic and therapeutic biomarker in hepatitis B virus-related hepatocellular carcinoma. *Int J Cancer.* 2022. doi:10.1002/ijc.34045

4. Hänzelmann S, Castelo R, Guinney J. GSVA: gene set variation analysis for microarray and RNA-seq data. *BMC bioinformatics.* 2013;14:7. doi:10.1186/1471-2105-14-7

5. Ayers M, Lunceford J, Nebozhyn M, et al. IFN-γ-related mRNA profile predicts clinical response to PD-1 blockade. *The Journal of clinical investigation.* 2017;127(8):2930-2940. doi:10.1172/jci91190

6. Rooney MS, Shukla SA, Wu CJ, Getz G, Hacohen N. Molecular and genetic properties of tumors associated with local immune cytolytic activity. *Cell.* 2015;160(1-2):48-61. doi:10.1016/j.cell.2014.12.033

7. Yoshihara K, Shahmoradgoli M, Martínez E, et al. Inferring tumour purity and stromal and immune cell admixture from expression data. *Nature communications.* 2013;4:2612. doi:10.1038/ncomms3612

8. Chen DS, Mellman I. Oncology meets immunology: the cancer-immunity cycle. *Immunity.* 2013;39(1):1-10. doi:10.1016/j.immuni.2013.07.012

9. Xu L, Deng C, Pang B, et al. TIP: A Web Server for Resolving Tumor Immunophenotype Profiling. *Cancer research.* 2018;78(23):6575-6580. doi:10.1158/0008-5472.Can-18-0689

10. Hong F, Meng Q, Zhang W, et al. Single-Cell Analysis of the Pan-Cancer Immune Microenvironment and scTIME Portal. *Cancer Immunol Res.* 2021;9(8):939-951. doi:10.1158/2326-6066.CIR-20-1026

11. Hoshida Y, Brunet J-P, Tamayo P, Golub TR, Mesirov JP. Subclass mapping: identifying common subtypes in independent disease data sets. *PLoS One.* 2007;2(11):e1195.

12. Roh W, Chen P-L, Reuben A, et al. Integrated molecular analysis of tumor biopsies on sequential CTLA-4 and PD-1 blockade reveals markers of response and resistance. *Sci Transl Med.* 2017;9(379). doi:10.1126/scitranslmed.aah3560
