# Supplementary material for: Guanylate‐binding proteins signature predicts favorable prognosis, immune‐hot microenvironment, and immunotherapy response in hepatocellular carcinoma
Source: Cancer Med. 2023 Aug 7;12(16):17504–21. doi: 10.1002/cam4.6347 (PMC10501289; doi:10.1002/cam4.6347)
Supplement: Supplementary file 4 — Table S5. Table S6. Table S7. Table S8. Table S10. Table S11. Table S12. [file CAM4-12-17504-s004.docx]

**Table S5. Baseline clinical characteristics of HCC patients in the TCGA cohort.**

| **Characteristics** | **N (%)** | **GBPs-Score**  **High Low** | | ***P***  **Chi squared test** |
| --- | --- | --- | --- | --- |
| **Total cases** | 369 (100%) | 185 | 184 |  |
| **Gender** |  |  |  | 0.070 |
| Male | 249 (67.5%) | 133 | 116 |  |
| Female | 120 (32.5%) | 52 | 68 |  |
| **Age** |  |  |  | 0.070 |
| <65 | 221 (59.9%) | 102 | 119 |  |
| ≥65 | 147 (39.8%) | 82 | 65 |  |
| **Histological type** |  |  |  | 0.133 |
| Fibrolamellar | 3 (0.8%) | 1 | 2 |  |
| Hepatocellular | 359 (97.3%) | 183 | 176 |  |
| Hepatocholangiocar | 7 (1.9%) | 1 | 6 |  |
| **History risk factors** |  |  |  | 0.083 |
| Alcohol consumption and/or NAFLD | 130 (35.2%) | 71 | 59 |  |
| Hemochromatosis | 5 (1.4%) | 3 | 2 |  |
| No risk history | 90 (24.4%) | 35 | 55 |  |
| Other | 125 (33.9%) | 68 | 57 |  |
| **Treatment** |  |  |  | 0.594 |
| Chemo-embolization | 26 (7.0%) | 14 | 12 |  |
| Radio-embolization | 6 (1.6%) | 4 | 2 |  |
| Chemo- and Radio-embolization | 4 (1.1%) | 1 | 3 |  |
| None | 17 (4.6%) | 10 | 7 |  |
| **Postoperative** |  |  |  | 0.715 |
| Yes | 15 (4.1%) | 9 | 6 |  |
| No | 223 (60.4%) | 123 | 100 |  |
| **Ablation embolization** |  |  |  | 0.235 |
| Yes | 13 (3.5%) | 9 | 4 |  |
| No | 233 (63.1%) | 122 | 111 |  |
| **Radiation therapy** |  |  |  | 0.232 |
| Yes | 4 (1.1%) | 1 | 3 |  |
| No | 240 (65.0%) | 132 | 108 |  |

Note: The marked in red are statistically significant.

**Table S6. Baseline clinical characteristics of HCC patients in the ICGC cohort.**

| **Characteristics** | **N (%)** | **GBPs-score**  **High Low** | | ***P***  **Chi squared test** |
| --- | --- | --- | --- | --- |
| **Total cases** | 231 (100%) | 116 | 115 |  |
| **Gender** |  |  |  | 0.220 |
| Male | 169 (72.8%) | 89 | 80 |  |
| Female | 62 (26.7%) | 27 | 35 |  |
| **Age** |  |  |  | 0.154 |
| <65 | 82 (35.3%) | 36 | 46 |  |
| ≥65 | 149 (64.2%) | 80 | 69 |  |
| **Alcohol intake** |  |  |  | 0.359 |
| No | 89 (38.4%) | 47 | 42 |  |
| Low | 33 (14.2%) | 19 | 14 |  |
| Medium | 61 (26.3%) | 25 | 36 |  |
| High | 33 (14.2%) | 15 | 18 |  |
| **Smoking** |  |  |  | 0.436 |
| Yes | 119 (51.3%) | 55 | 64 |  |
| No | 99 (42.7%) | 51 | 48 |  |
| **Virus infection** |  |  |  | 0.314 |
| HBV | 59 (25.4%) | 27 | 32 |  |
| HCV | 122 (52.6%) | 68 | 54 |  |
| HBV and HCV | 4 (1.7%) | 2 | 2 |  |
| None | 42 (18.1%) | 17 | 25 |  |

Note: Those marked in red are statistically significant.

**Table S7. Baseline clinical characteristics of HCC patients in the CHCC cohort.**

| **Characteristics** | **N (%)** | **GBPs-score**  **High Low** | | ***P***  **Chi squared test** |
| --- | --- | --- | --- | --- |
| **Total cases** | 159 (100%) | 79 | 80 |  |
| **Gender** |  |  |  | 0.173 |
| Male | 128 (80.5%) | 67 | 61 |  |
| Female | 31 (19.5%) | 12 | 19 |  |
| **Age** |  |  |  | 0.159 |
| <65 | 137 (86.2%) | 65 | 72 |  |
| ≥65 | 22 (13.8%) | 14 | 8 |  |
| **Tumor encapsulation** |  |  |  | 0.457 |
| Complete | 111 (69.8%) | 53 | 58 |  |
| No | 48 (30.2%) | 26 | 22 |  |
| **Tumor thrombus** |  |  |  | 0.603 |
| Yes | 37 (23.3%) | 17 | 20 |  |
| No | 122 (76.7%) | 62 | 60 |  |

Note: Those marked in red are statistically significant.

**Table S8. Baseline clinical characteristics of HCC patients in the GSE76427.**

| **Characteristics** | **N (%)** | **GBPs-score**  **High Low** | | ***P***  **Chi squared test** | |
| --- | --- | --- | --- | --- | --- |
| **Total cases** | 115 (100%) | 58 | 57 |  |  |
| **Gender** |  |  |  | 0.668 |  |
| Male | 93 (80.9%) | 46 | 47 |  |  |
| Female | 22 (19.1%) | 12 | 10 |  |  |
| **Age** |  |  |  | 0.928 |  |
| <65 | 59 (51.3%) | 30 | 29 |  |  |
| ≥65 | 56 (48.7%) | 28 | 28 |  |  |

Note: Those marked in red are statistically significant.

**Table S10. Correlation of clinicopathologic characteristics and GBPs-score in the ICGC cohort.**

| **Characteristics** | **N (%)** | **GBPs-score**  **High Low** | | ***P***  **Chi squared test** | |
| --- | --- | --- | --- | --- | --- |
| **Total cases** | 231 (100%) | 116 | 115 |  |  |
| **Stage** |  |  |  | 0.071 |  |
| I-II | 139 (60.2%) | 76 | 63 |  |  |
| III-IV | 83 (35.9%) | 35 | 48 |  |  |
| **T-stage** |  |  |  | 0.101 |  |
| T1-2 | 142 (61.5%) | 77 | 65 |  |  |
| T3-4 | 86 (37.2%) | 37 | 49 |  |  |
| **N-stage** |  |  |  | 0.080 |  |
| N0 | 224 (97.0%) | 114 | 110 |  |  |
| N1 | 3 (0.9%) | 0 | 3 |  |  |
| **M-stage** |  |  |  | 0.316 |  |
| M0 | 227 (98.3%) | 113 | 114 |  |  |
| M1 | 1 (0.4%) | 0 | 1 |  |  |
| **Grade** |  |  |  | 0.008 |  |
| G1-2 | 148 (64.1%) | 86 | 62 |  |  |
| G3-4 | 54 (23.4%) | 20 | 34 |  |  |
| **Tumor size** |  |  |  | 0.001 |  |
| <5cm | 179 (77.5%) | 101 | 78 |  |  |
| 5-10cm | 37 (16.0%) | 10 | 27 |  |  |
| ≥10cm | 12 (5.2%) | 3 | 9 |  |  |
| **Portal vein invasion** |  |  |  | 0.003 |  |
| No | 166 (71.9%) | 93 | 73 |  |  |
| Yes | 62 (26.8%) | 21 | 41 |  |  |
| **Hepatic vein invasion** |  |  |  | 0.014 |  |
| No | 181 (78.4%) | 98 | 83 |  |  |
| Yes | 47 (20.3%) | 16 | 31 |  |  |
| **Hepatic artery invasion** |  |  |  | 0.081 |  |
| No | 225 (97.4%) | 114 | 111 |  |  |
| Yes | 3 (1.3%) | 0 | 3 |  |  |
| **Bile duct invasion** |  |  |  | 0.031 |  |
| No | 204 (88.3%) | 107 | 97 |  |  |
| Yes | 24 (10.4%) | 7 | 17 |  |  |
| **Liver fibrosis score** |  |  |  | 0.186 |  |
| 0 | 20 (8.7%) | 7 | 13 |  |  |
| 1-2 | 76 (32.9%) | 35 | 41 |  |  |
| 3-4 | 132 (57.1%) | 72 | 60 |  |  |

Note: Those marked in red are statistically significant.

**Table S11. Correlation of clinicopathologic characteristics and GBPs-Score in the TCGA cohort.**

| **Characteristics** | **N (%)** | **PR-Score**  **High Low** | | ***P***  **Chi squared test** | |
| --- | --- | --- | --- | --- | --- |
| **Total cases** | 369 (100%) | 185 | 184 |  |  |
| **Stage** |  |  |  | <0.001 |  |
| I-II | 257 (69.6%) | 143 | 114 |  |  |
| III-IV | 88 (23.8%) | 28 | 60 |  |  |
| **T-stage** |  |  |  | <0.001 |  |
| T1 | 181 (49.1%) | 105 | 76 |  |  |
| T2 | 94 (25.5%) | 46 | 48 |  |  |
| T3 | 78 (21.1%) | 22 | 56 |  |  |
| T4 | 13 (3.5%) | 9 | 4 |  |  |
| **M-stage** |  |  |  | 0.015 |  |
| M0 | 265 (71.8%) | 122 | 143 |  |  |
| M1 | 4 (1.1%) | 1 | 3 |  |  |
| Mx | 100 (27.1%) | 62 | 38 |  |  |
| **N-stage** |  |  |  | 0.014 |  |
| N0 | 250 (67.8%) | 117 | 133 |  |  |
| N1 | 4 (1.1%) | 0 | 4 |  |  |
| Nx | 114 (30.9%) | 67 | 47 |  |  |
| **Grade** |  |  |  | 0.029 |  |
| G1-2 | 232 (62.9%) | 126 | 106 |  |  |
| G3-4 | 132 (35.8%) | 56 | 76 |  |  |
| **Fibrosis score** |  |  |  | 0.608 |  |
| 0-no fibrosis | 74 (20.1%) | 37 | 37 |  |  |
| 1-4 fibrosis | 58 (8.4%) | 29 | 29 |  |  |
| 5-6 cirrhosis | 77 (19.0%) | 44 | 33 |  |  |
| **Fetoprotein value** |  |  |  | <0.001 |  |
| <400 | 213 (57.7%) | 126 | 87 |  |  |
| ≥400 | 65 (17.6%) | 20 | 45 |  |  |
| **Vascular invasion type** |  |  |  | 0.047 |  |
| Macro | 14 | 4 | 10 |  |  |
| Micro | 90 | 38 | 52 |  |  |
| None | 205 | 111 | 94 |  |  |
| **Inflammation extent type** |  |  |  | 0.869 |  |
| Severe | 17 | 52 | 46 |  |  |
| Mild | 98 | 58 | 59 |  |  |
| None | 117 | 9 | 8 |  |  |

Note: Those marked in red are statistically significant.

**Table S12. Correlation of clinicopathologic characteristics and GBPs-score in the CHCC cohort.**

| **Characteristics** | **N (%)** | **PR-Score**  **High Low** | | ***P***  **Chi squared test** | |
| --- | --- | --- | --- | --- | --- |
| **Total cases** | 159 (100%) | 79 | 80 |  |  |
| **Stage** |  |  |  | 0.955 |  |
| I-II | 105 (66.0%) | 52 | 53 |  |  |
| III-IV | 54 (34.0%) | 27 | 27 |  |  |
| **BCLC Stage** |  |  |  | 0.680 |  |
| A | 68 (42.8%) | 35 | 33 |  |  |
| B | 52 (32.7%) | 27 | 25 |  |  |
| C | 39 (24.5%) | 17 | 22 |  |  |
| **Tumor number** |  |  |  | 0.137 |  |
| 1 | 117 (73.6%) | 54 | 63 |  |  |
| >1 | 42 (26.4%) | 25 | 17 |  |  |
| **Tumor size** |  |  |  | 0.326 |  |
| <5cm | 76 (47.8%) | 40 | 36 |  |  |
| 5-10cm | 57 (35.8%) | 24 | 33 |  |  |
| >10cm | 26 (16.4%) | 15 | 11 |  |  |
| **Liver fibrosis** |  |  |  | 0.357 |  |
| Yes | 112 (70.4%) | 53 | 59 |  |  |
| No | 47 (29.6%) | 26 | 21 |  |  |
| **Lymph node metastasis** |  |  |  | 0.157 |  |
| Yes | 2 (1.3%) | 0 | 2 |  |  |
| No | 157 (98.7%) | 79 | 78 |  |  |
| **AFP** |  |  |  | <0.001 |  |
| <400 | 58 (36.5%) | 17 | 41 |  |  |
| ≥400 | 101 (63.5%) | 62 | 39 |  |  |

Note: Those marked in red are statistically significant.
